# Pharmacological Properties and Molecular Mechanisms of Thymol: Prospects for Its Therapeutic Potential and Pharmaceutical Development

**DOI:** 10.3389/fphar.2017.00380

**Published:** 2017-06-26

**Authors:** Mohamed Fizur Nagoor Meeran, Hayate Javed, Hasan Al Taee, Sheikh Azimullah, Shreesh K. Ojha

**Affiliations:** ^1^Department of Pharmacology and Therapeutics, College of Medicine and Health Science, United Arab Emirates UniversityAl Ain, United Arab Emirates; ^2^Department of Biochemistry, College of Medicine and Health Science, United Arab Emirates UniversityAl Ain, United Arab Emirates

**Keywords:** thymol, antioxidant, free radical scavenger, cancer, animals, drug discovery, phytochemicals, natural compounds

## Abstract

Thymol, chemically known as 2-isopropyl-5-methylphenol is a colorless crystalline monoterpene phenol. It is one of the most important dietary constituents in thyme species. For centuries, it has been used in traditional medicine and has been shown to possess various pharmacological properties including antioxidant, free radical scavenging, anti-inflammatory, analgesic, antispasmodic, antibacterial, antifungal, antiseptic and antitumor activities. The present article presents a detailed review of the scientific literature which reveals the pharmacological properties of thymol and its multiple therapeutic actions against various cardiovascular, neurological, rheumatological, gastrointestinal, metabolic and malignant diseases at both biochemical and molecular levels. The noteworthy effects of thymol are largely attributed to its anti-inflammatory (*via* inhibiting recruitment of cytokines and chemokines), antioxidant (*via* scavenging of free radicals, enhancing the endogenous enzymatic and non-enzymatic antioxidants and chelation of metal ions), antihyperlipidemic (*via* increasing the levels of high density lipoprotein cholesterol and decreasing the levels of low density lipoprotein cholesterol and low density lipoprotein cholesterol in the circulation and membrane stabilization) (*via* maintaining ionic homeostasis) effects. This review presents an overview of the current *in vitro* and *in vivo* data supporting thymol’s therapeutic activity and the challenges concerning its use for prevention and its therapeutic value as a dietary supplement or as a pharmacological agent or as an adjuvant along with current therapeutic agents for the treatment of various diseases. It is one of the potential candidates of natural origin that has shown promising therapeutic potential, pharmacological properties and molecular mechanisms as well as pharmacokinetic properties for the pharmaceutical development of thymol.

## Introduction

Extensive epidemiological and experimental studies have suggested the health benefits of medicinal herbs as well as their constituents against various human ailments (Sofowora et al., 2013). The therapeutic importance of plants have been quoted in the ancient cultures and traditions of many countries and societies and they are believed to be cost effective and safe. Since ancient times, plants and their products are used as a culinary preparation or as a remedy in different traditional medicine for many diseases (Rahmani et al., 2014). Turmeric, oregano, thyme, olives and dates, to name a few, have been used extensively for culinary purposes in diets and are also believed to possess beneficial effects against numerous diseases (Rahmani et al., 2014). Among these spices, the Greeks, Romans, and Egyptians have used thyme as a preservative, odorant and flavoring agent in foods. It is a small subshrub abundantly used as a traditional medicine in the western Mediterranean region and its leaves are often used as herbal medicinal products and food additives (Zarzuelo and Crespo, 2002). Thyme possesses potent antibacterial, antifungal, sedative, antiseptic, antioxidative, expectorant, antispasmodic, antifungal, antivirotic, antihelminthic, carminative and diaphoretic effects (Rustaiyan et al., 2000; Soliman and Badeaa, 2002). Thyme contains abundant amount of terpenoids, flavonoids, glycosides and phenolic acids (Vila, 2002).

Among many constituents, thymol, chemically known as 2-isopropyl-5-methylphenol is a dietary monoterpene phenol and is abundantly found in certain plants such as *Thymus vulgaris* (Amiri, 2012), *Ocimum gratissimum* (Pandey et al., 2014), *Thymus ciliates* (Kabouche et al., 2009), *Satureja thymbra* (Markovic et al., 2011), *Thymus* zygis (Ocana and Reglero, 2012), *Trachyspermum ammi* (Bairwa et al., 2012), *Carum copticum* (Khajeh et al., 2004), *Satureja intermedia* (Yousefzadi et al., 2012), *Thymbra capitata* (Miguel et al., 2015), *Lippia multiflora* (Ku and Lin, 2013), *Thymus pectinatus* (Vardar-Unlu et al., 2003), *Zataria multiflora* (Veras et al., 2013), *Satureja hortensis* (Soran and Lung, 2010), *Centipeda minima* (Liang et al., 2007) and *Nigella sativa* seeds (Ghosheh et al., 1999). Thymol possesses antibacterial (Didri et al., 1994), antifungal (Mahmoud, 1994), anti-inflammatory (Aeschbach et al., 1994), antioxidant (Yanishlieva et al., 1999), anti-mutagenic (Zahin et al., 2010), larvicidal (Pavela, 2009), analgesic (Ozen et al., 2011), anti-microbial (Karpanen et al., 2008), acaricidal (Araujo et al., 2015), anticonvulsant, antiepileptogenic (Sancheti et al., 2014), wound healing (Riella et al., 2012), anti-hemolytic (Alinezhad et al., 2013), antiphlogistic (Anamura, 1989), antileishmanial (Robledo et al., 2005) and radioprotective (Archana et al., 2011a) properties. Thymol improves digestion by relaxing smooth muscles, prevents menstrual cramps, attenuates respiratory problems and is an active ingredient used in food flavorings, topical ointments, various soaps, toothpastes, shampoos, deodorants and mouthwashes (Shapiro et al., 1994; Manou et al., 1998). Due to its potent antimicrobial properties, thymol is frequently used in dentistry for the treatment of oral cavity infections (Maruniak et al., 1992; Shapiro and Guggenheim, 1995; Twetman et al., 1995; Ogaard et al., 1997; Khan et al., 2017). These pharmacological properties of thymol are ascribed to the pharmacophore of the phenolic hydroxyl group in its chemical structure. The compounds containing the phenolic groups are known to confer protection against the deleterious effects of free radicals both by absorbing or neutralizing free radicals and by augmenting endogenous antioxidants (Wojdylo et al., 2007).

Thymol is biosynthesized by the hydroxylation of *p*-cymene after the aromatization of γ-terpinene to *p*-cymene (Poulose and Croteau, 1978). The lethal dose (LD_50_) value of thymol for rats and guinea pigs is 980 mg/kg and 88 mg/kg, body weight, respectively (Jenner et al., 1964). For male and female ddY mice the LD_50_ is 1200 and 1050 mg/kg, respectively (Hasegawa et al., 1989), whereas the LD_50_ for cats, rabbits and mice are 250 mg/kg, 750 mg/kg and 640 mg/kg, respectively (Instituto Superiore di Sanita, 1999). The time-tested safety and activity of thymol can also be attested to by the use of thymol for centuries in different cultures and civilizations. One example is the use of thymol containing herbs by the ancient Egyptians for the preservation of mummies. According to the Environmental Protection Agency, there are no known adverse effects with respect to thymol when used in animals and humans. Thymol is cataloged by the United States Food and Drug Administration as ‘Generally Recognized As Safe’ for use as a food additive, therefore it is considered to be safe with negligible toxicity.

An extensive literature survey has revealed some excellent reviews on essential oils regarding their phytochemical and pharmacological activities based on their various pharmacological properties. There is a short review describing the therapeutic and pharmacological effects of thymol (Parasei et al., 2016) and a detailed one presenting the antimicrobial properties of thymol (Marchese et al., 2016). But there are no reviews that have been published focusing on the multi-pharmacological properties of thymol against various human ailments until now. Thus, thist review aims to reveal the various pharmacological activities and therapeutic potential of thymol as well as prospects for its pharmaceutical development followed by its mechanism of action demonstrated in both *in vivo* and *in vitro* studies.

### Chemistry and Physical Characteristics of Thymol

Thymol (2-isopropyl-5-methylphenol) is a white crystalline substance that gives thyme its strong flavor, pleasant aromatic odor and strong antiseptic property. Its density at 25°C is 0.96 g/cm^3^ with a melting point ranging from 49°C to 51°C (322-324 K; 120-124°F) (Jordan et al., 1991; Lide and Frederikse, 1996). It is highly soluble in alcohols, alkaline solutions and other organic solvents due to the deprotonation of phenol but it is slightly soluble in water at neutral pH and it absorbs maximum UV radiation at 274 nm (Norwitz et al., 1986; Wade and Reynolds, 1997). It has low solubility in water and its unpleasant taste and smell makes it less palatable (Nieddu et al., 2014). It also has low solubility in the hydrophobic domain of the bacterial cytoplasmic membrane due to its hydrophobicity (Trombetta et al., 2005). For the past few decades, the synthesis of thymol has been achieved using the reaction between *p*-cymene and *m*-cresol with isopropyl alcohol or propene and by the use of supercritical CO_2_ (Amandi et al., 2005; Nagle et al., 2013). Thymol is an important agent of natural origin and has generated interest in the scientific community in pharmacological studies for its therapeutic potential in different diseases. The present review presents an overview of its preclinical data, pharmacokinetics, pharmacological properties and therapeutic potential in different human diseases.

### Pharmacokinetics of Thymol

#### Absorption

Previous reports have revealed the rapid absorption of thymol following oral administration and its degradation in the stomach or intestine (Michiels et al., 2008; Anderson et al., 2012). A report from Schroder and Vollmer (1932) has evidenced the presence of thymol in the stomach, intestine, and urine after its oral administration with sesame oil at a dose around 500 mg in rats and 1–3 g in rabbits. A single dose of thymol (1 or 3 g) encapsulated in gelatin capsule administered to dogs showed the presence of thymol conjugates in urine (22 or 34%) after 3–4 h following urine and fecal analysis (Robbins, 1934). Oral administration of a single dose of thymol (50 mg/kg) was rapidly absorbed and slowly eliminated approximately within 24 h (Nieddu et al., 2014). The maximum concentration (T_max_) was reached after 30 min, while approximately 0.3 h was needed for the half-life of the absorption phase (*t*_1/2_). The lower concentrations of thymol were recovered in the liver, lungs, kidneys, and muscles while its higher concentrations were detected in the mucosa and other inner contents of the intestines indicating its partial absorption (Nieddu et al., 2014). According to the report of Kohlert et al. (2002), after the intake of one Bronchipret^®^ TP tablet that is equivalent to 1.08 mg of thymol, the plasma concentrations of thymol metabolites were detectable after 20 min. The rapid absorption of thymol indicates that it’s mainly absorbed in the upper component of the gut. In healthy volunteers, the oral administration of one Bronchipret^®^ TP tablet resulted in a peak plasma concentration (C_max_) of 93.11 ng/ml, T_max_ of 1.97 h, *t*_1/2_ of 10.2 h, area under time curve from time 0 to clast (AUC_0→clast_ of 837.3 ngh/ml, mean residence time after extravascular administration (MRT_abs_) of 12.6 h and a mean absorption time (MAT) of 0.53 h (Kohlert et al., 2002). It also had a total body clearance of 1.2 l/h, volume of distribution at steady state of 14.7 l and a volume of distribution during the elimination phase of 17.7 h was reported in healthy humans (Kohlert et al., 2002).

#### Distribution

Free thymol is usually not detectable in human plasma. It is circulated as thymol sulfate, not glucuronide, in the blood stream as detected by liquid chromatography-mass spectrophotometry/mass spectrophotometry (LC-MS/MS). Thymol sulfate has been detected in plasma 20 min after administration. The maximum plasma levels (93.1 ± 24.5 ng/ml) of thymol were reported after 1.97 ± 0.77 h of administration. After administration the bioavailability of thymol measured in plasma as thymol sulfate was found to be about 16%. It is eliminated by kidneys and is measured in the urine as thymol conjugates. The volume of distribution (Vdss/f) of 14.7 L has revealed that thymol sulfate mainly resides in the extracellular space (Kohlert et al., 2002).

#### Metabolism

Thymol undergoes glucuronidation by uridine 5′-diphospho-glucuronosyltransferase (UGT) following secretion into the proximal tubule (Raoof et al., 1996; Shipkova et al., 2001). The absence of thymol glucuronide in plasma could be due to the lower activity of hepatic UGT compared to sulfotransferase and the formation of glucuronide was shown only at much higher doses (Ogata et al., 1995). In healthy human volunteers, thymol (0.6 g/kg) was metabolized to thymol sulfate, thymol glucronide and thymol thymohydroquinone sulfate and it was excreted in urine (Takada et al., 1979).

#### Elimination

The elimination of thymol conjugates in urine was detectable for the first 24 h, with the majority being eliminated after 6 h. The combined amount of both thymol sulfate and glucuronide excreted in urine during the first 24 h was 16.2 ± 4.5% of thymol intake. The renal clearance was calculated to be 0.271 ± 0.7 L/h (Kohlert et al., 2002). Takada et al. (1979) studied the metabolism of thymol in rabbits and humans, wherein thymol (0.5 g/kg) fed to rabbits metabolized to thymol glucuronide as the main metabolite of thymol and eliminated as glucuronic acid and sulfuric acid metabolites (Takada et al., 1979). Austgulen et al. (1987) reported the rapid excretion of thymol and its metabolites in the urine of male albino Wistar rats after thymol was dosed by gavage (1 mM/kg) as analyzed by capillary gas chromatography–mass spectrometry (GC-MS). Williams (1959) summarized the previous reports on the metabolism of thymol and reported its excretion as sulfate and glucuronide conjugates in the urine of dogs, rats and humans. Around 1g (one third) of the dose was excreted in the urine of dogs while feces were found to be devoid of thymol (Robbins, 1934).

### Pharmacological Properties of Thymol

#### Antioxidant Properties

The antioxidant properties of thymol have been well documented in various preclinical studies including cell lines and animal models. At high rate constants, it effectively scavenged the hydroxyl free radicals thereby producing major transient species named phenoxyl radicals. The generated adducts from the phenoxyl radicals undergo dehydration which can be accelerated by an alkaline medium. The addition of hydroxyl radicals at the ortho position (C6 atom) of the phenolic group yields the phenoxyl radical after dehydration. The attack at the ortho position is more favorable energetically while the attack at the para position is also expected to occur. Furthermore, additions at the ortho positions occur without any precomplex formation. The non-toxicity and redox potential of the thymol•/thymol couple makes it a promising antioxidant (Venu et al., 2013).

One of the most studied effects of thymol includes the scavenging of free radicals by increasing the activities of several endogenous antioxidant enzymes levels *viz.* superoxide dismutase (SOD), catalase, glutathione peroxidase (GPx), glutathione-*S*-transferase (GST) along with non-enzymatic antioxidants such as vitamin C, vitamin E and reduced glutathione (GSH) (Nagoor Meeran and Prince, 2012). A comparative study revealed that thymol has superior reducing power, DPPH, superoxide and hydroxyl radical scavenging activity and bestows protection against oxidative damage to lipids (Nagoor Meeran et al., 2015b). The various supportive evidences for the antioxidant activity of thymol are detailed below.

#### *In Vitro* Studies

Thymol was shown to exhibit potent superoxide anion, hydroxyl and DPPH radical scavenging and reducing capacity in a concentration dependent manner (Nagoor Meeran and Prince, 2012; Nagoor Meeran et al., 2015b, 2016b). Thymol possesses SOD like activity in removing superoxide radicals *in vitro* (Kruk et al., 2000). It has also shown moderate antioxidant activity in V79 Chinese hamster lung fibroblast cells (Undeger et al., 2009). Thymol (25 μg/ml) showed potent antioxidant activity by modulating the activities of enzymatic antioxidants and decreased lipid peroxidation in gamma ray induced V79 Chinese hamster cells (Archana et al., 2011b). Thymol (0.02–0.20%) showed better antioxidant capacity than its isomer carvacrol in lipid systems due to its greater steric hindrance and it was shown that at room temperature it inhibited the autoxidation of two lipid systems namely triacylglycerols of lard (TGL) and triacylglycerols of sunflower oil (TGSO). Furthermore, it also inhibited the oxidation of TGL and TGSO, with potent antioxidant activity against TGSO. Thymol increased chain initiation during the oxidation of TGSO more so than during the oxidation of TGL (Yanishlieva et al., 1999). In the intestinal Caco-2 cell line, thymol (250 μM) attenuated oxidative stress induced by hydrogen peroxide (H_2_O_2_) (Cabello et al., 2015). Thymol (250 and 500 μM) strongly inhibited nicotinamide adenine dinucleotide phosphate reduced (NADPH)-cytochrome-c reductase mediated lipid peroxidation isolated from detergent solubilized liver microsomes of rats (Kamataki et al., 1978). Thymol attenuated the production of reactive oxygen species (ROS) and showed myeloperoxidase inhibitory activity in human neutrophils (Perez-Roses et al., 2016).

#### *In Vivo* Studies

Owing to its potent antioxidant potential, thymol showed radioprotective and anticlastogenic potential in gamma radiation induced Swiss albino mice (Archana et al., 2011b). Thymol supplementation increased the antioxidant status and decreased malondialdehyde (MDA) levels in broiler chickens (Zidan et al., 2016). Dietary supplementation with the combination of carvacrol–thymol (1:1) (100 mg/kg) reduced the occurrence of oxidative stress and the impairment of the intestinal barrier in weaning piglets by its potent antioxidant property (Wei et al., 2016). Thymol (24.7 mg/kg) attenuated aflatoxin-induced oxidative stress in male rats due to its potent antioxidant activity (El-Nekeety et al., 2011). Thymol (7.5 mg/kg) has been shown to inhibit lipid peroxidation, glycation, dyslipidemia, inflammation, ionic homeostasis malfunction and apoptosis by virtue of its potent antioxidant property (Nagoor Meeran and Prince, 2012; Nagoor Meeran et al., 2014, 2015b,c, 2016b).

#### Anti-inflammatory Properties

Thymol (150 μM) has been shown to ameliorate LPS-induced inflammation in murine macrophage cell lines (Chauhan et al., 2014). Thymol (84 μg/ml) treatment attenuated lipopolysaccharide (LPS) and interferon gamma (IFN-γ) induced macrophage inflammation *in vitro* by inhibiting messenger RNA expression of inducible nitric oxide (NO) in J774A.1 cell lines (Vigo et al., 2004). In human polymorphonuclear neutrophils (PMNs), thymol (10 and 20 μg/ml) inhibited the synthetic chemotactic peptide *N*-formyl-methionyl-leucyl-phenylalanine (fMLP)-induced release of elastase, a marker of inflammatory diseases and a serine proteinase released by activated human neutrophils in a concentration-dependent manner (Braga et al., 2006). Thymol (100 μM) has been reported to alter prostaglandin catalyzed biosynthesis by inhibiting both isoforms of cyclooxygenase (COX), with the most active being against COX-1 with an IC_50_ value of 0.2 μM. These studies suggest the potential of thymol as an anti-inflammatory drug and indicate that it could be used in a similar fashion to non-steroidal anti-inflammatory drugs (Marsik et al., 2005). Thymol (1.1 μg/ml) exhibited inhibitory effects against arachidonic-acid-induced blood coagulation and platelet aggregation *in vitro* (Enomoto et al., 2001). Thymol (50–150 μM) attenuated bleomycin induced genotoxicity in human ovarian cells (SKOV-3) by virtue of its antioxidant and anti-inflammatory properties (Arab et al., 2015). Ku and Lin (2013) reported the anti-inflammatory nature of thymol by its inhibiting of the T cell immune response and improved T-helper cells-1 (Th1) (interleukin-2 (IL-2) and IFN-γ/T-helper cells-2 (Th2) (interleukin-4 (IL-4), interleukin-5 (IL-5) and interleukin-10 (IL-10) ratio in mouse primary splenocytes. Thymol (40 μg/ml) inhibited the LPS stimulated inflammatory response in mouse mammary epithelial cells mediating the down regulation of mitogen-activated protein kinases (MAPK) and nuclear factor-kB (NF-κB) signaling pathways (Liang et al., 2014).

Thymol (7.5 mg/kg) abrogated the induction of inflammation in isoproterenol (ISO) challenged rats, an animal model of myocardial infraction (MI), which had developed myocardial necrosis. (Nagoor Meeran et al., 2015b). Thymol isolated from essential oils of *Lippia gracilis* leaves has been shown to inhibit carrageenan-induced edema formation in the paws (administered at the dose of 200 mg/kg) similar to the activity of positive control acetylsalicylic acid (300 mg/kg) (Mendes et al., 2010). Furthermore, treatment with this essential oil at the dose of 50, 100, and 200 mg/kg abrogated leukocyte migration into the peritoneal cavity in carrageenan-challenged experimental animals. Treatment with the essential oil also inhibited the occurrence of abdominal writhes in experimental animals induced by acetic acid.

Thymol isolated from leaf essential oils of *Lippia gracilis* (32.68%) is believed to be primarily responsible for its antinociceptive and anti-inflammatory actions (Mendes et al., 2010). It has been shown to inhibit the release of arachidonic acid, COX and the biosynthesis of prostaglandins such as prostaglandin E2 (PGE2) in the visceral pain model (Mendes et al., 2010). Monteiro et al. (2007) has demonstrated the anti-inflammatory effect of thymol present in the *Lippia sidoides* essential oil administered at the doses of 1 and 10 mg/ear in the acute ear edema model induced by 12-*O*-tetradecanoyl phorbol 13-acetate (TPA) in mice as evidenced by reduced edema (a 45.93 and 35.26% reduction, respectively). Thymol (100 mg/kg) attenuated inflammation and promoted wound healing in several rodent models *via* inhibiting the influx of leucocytes to the injured areas and thus preventing edema (Riella et al., 2012). Thymol exhibited potent anti-inflammatory activity by diminishing the release of inflammatory mediators *viz.* prostanoids, interleukins and leukotrienes in the buccal sites of young adults (Skold et al., 1998; Yucel-Lindberg et al., 1999). Thymol (10–250 μg/pellet) also elicited potent anti-inflammatory and antiangiogenic action in chorioallantoic membrane (CAM) assay using the experiment model of CAM of the fertilized hen’s egg (Demirci et al., 2004).

Thymol (50 μg/ml) increased the mean fluorescence intensity (MFI) of cluster of differentiation 40 (CD40), cluster of differentiation 86 (CD86) and major histocompatibility complex-II (MHCII) expressions determined by flow cytometric analysis in the dendritic cells isolated from spleen of BALB/c mice (Amirghofran et al., 2016). Thymol inhibited ROS (IC_50_= 3 μg/ml), reactive nitrogen species (RNS) (IC_50_= 4.7) and significantly reduced generation of NO and H_2_O_2_ as well as activities of nitric oxide synthase (NOS) and nicotinamide adenine dinucleotide reduced oxidase (NADH oxidase) in LPS-induced murine macrophages (Kavoosi et al., 2012). A report from Hejazian (2006) has revealed that thymol present as an important constituent in the essential oil of *Carum copticum* (20 mg/kg) significantly diminished pain sensation in the inflammatory phase of the formalin test in mice. Lorente et al. (1989) revealed an important pharmacological advantage of the potentiation of anti-inflammatory activity of α and β-pinene mixtures (80 mg/kg) upon use in conjunction with thymol (1 mg/kg) in female Wistar rats. Thymol (10 and 20 μg/ml) reduced inflammatory responses through modulation of the expression of c-Jun N-terminal kinase (JNK), stress-activated protein kinases (STAT-3), activator protein-1 (AP-1) and nuclear factors of activated T-cells (NFATs) in LPS treated macrophages (Gholijani et al., 2016). Thymol (600 μM) reduced immunoglobulin-E (IgE)-dependent responses in mast cells through the activation of apoptotic cell death in bone marrow-derived mast cells (BMMCs) and BALB/c mice (Wechsler et al., 2014).

#### Immunological Properties

Thymol (25–200 mg/kg) was shown to modulate the immune system in cyclosporine-A treated Swiss albino mice by enhancing the expressions of cluster of differentiation 4 (CD4), cluster of differentiation 8 (CD8) and Th1 cytokines via upregulation of IFN-γ expression and enhanced secretion of interleukin-12 (IL-12) (Chauhan et al., 2010). Khajeali et al. (2012) reported that thymol produced a significant increase in antibody titers against the Newcastle disease virus in broiler chickens. Thymol feed supplementation (0–200 mg/kg) improved the activities of digestive enzymes, growth indices and antioxidant status with declined MDA levels. It also improved the immune response via increasing tolerance to hypersensitivity and immunoglobulin G (IgG) in broiler chickens (Hashemipour et al., 2013). It improved innate immunity (Giannenas et al., 2012). Thymol diet supplementation (1%) enhanced the levels of immunoglobulin A (IgA) and immunoglobulin M (IgM) in the sera of weaning pigs challenged with *Salmonella typhimurium* (Trevisi et al., 2010). Thymol treatment in low-weight growing-finishing pigs raised the percentage of CD4+, CD8+, and MHC-II in their peripheral blood and it also down regulated tumor necrosis factor-α (TNF-α) expression in the stomach of post-weaned pigs (Taranu et al., 2012).

Thymol (IC_50_= 7.69 μg/ml) suppressed the adhesion and superoxide production on isolated ovine neutrophils and also anti-inflammatory property on sheep neutrophils (Farinacci et al., 2008). Thymol has been shown to improve the immune system by increasing the levels of IgA and IgM in the pig’s gut (Li et al., 2012). Thymol (10 μg/ml) attenuated the maturation of dendritic cells and inhibited the mitogenic and allogenic T cell responses along with the secretion of IFN-γ and IL-4 cytokines (Amirghofran et al., 2016). Thymol (10 μg/ml) increased phagocytosis by enhancing the membrane fluidity of macrophages and suppressed the inflammatory responses by downregulating the secretion of pro-inflammatory cytokines by its potent immunostimulating effect (Chauhan et al., 2014).

#### Antimicrobial Properties

There are a convincing number of studies that reveal that thymol alone or thymol in plants along with other metabolites possess potent antimicrobial, antifungal, antibacterial, and antiparasitic properties. Marchese et al. (2016) has comprehensively reviewed the antimicrobial properties of thymol. Thymol (32.55%) present in the essential oil of *Thymus vulgaris* L. showed bacteriostatic activity against most of the gram positive and negative bacteria (Marino et al., 1999). A report from Olasupo et al. (2003) revealed the antibacterial effect of thymol with the lowest minimum inhibitory concentration (MIC) values of 1.0 mmol/L (*S. typhimurium*) and 1.2 mmol/L (*Escherichia coli*). Thymol possesses antimicrobial activity against *S. aureus* (MIC: 0.31 mg/ml) and *E. coli* (MIC: 5.00 mg/ ml) by the perturbation of the lipid fraction of the bacterial plasma membrane resulting in the leakage of intracellular materials (Trombetta et al., 2005). The thymol chemotypte of the essential oils of *T. zygis* and *T. vulgaris* has shown antibacterial effect against various gram negative and positive bacterial strains with MIC ≤ 0.2 μl/ml (Rota et al., 2008). Thymol (200 mg/ml) could inhibit the growth of *E. coli*. by inducing the permeabilization and depolarization of the cytoplasmic membrane (Xu et al., 2008). Thymol at 2.5 mM inhibits the growth of *S. aureus, E. coli* and *S. typhimurium*. Furthermore, a synergistic interaction was found for thymol with all antibiotics tested against *E. coli, S. typhimurium, S. aureus*, and *S. pyogenes* (Palaniappan and Holley, 2010). Thymol ester derivatives were found to be more effective against streptococcus species (Mathela et al., 2010). Thymol was found to possess antibacterial activity against selected verocytotoxigenic *E. coli* (Rivas et al., 2010). Thymol (0.12%) possess antifungal activity against *C. albicans* MTCC 227 biofilm inhibition (Pemmaraju et al., 2013). Gelatin films containing different thymol concentrations (1–8%) produced inhibitory zones ranging from 30 to 46 mm against several bacteria. Thymol was more effective against Gram positive strains (Kavoosi et al., 2013). Thymol (15 and 30 mg/kg) was shown to possess cytotoxic and antileishmanial activities in hamsters experimentally infected with *Leishmania* (*Viannia*) *panamensis* (Robledo et al., 2005). Thymol derivative named benzoyl-thymol was the best inhibitor (8.67 ± 0.28 μg/mL) against *Leishmania infantum chagasi* (de Morais et al., 2014).

#### Other Pharmacological Properties

The blocking effect of thymol on voltage-activated sodium currents has been investigated in the *in vitro* setup using experimental cell models of animal and human origin. For skeletal muscle and the neuronal sodium channel, it showed a half-maximum blocking concentration (IC_50_) of 104 and 149 μM, respectively. Blockade of voltage-operated sodium channels were attributed to confer the antinociceptive and anesthetic effects (Haeseler et al., 2002). In rat skeletal muscle fibers isolated enzymatically, thymol (30–600 μg) treatment suppressed both calcium (Ca^2+^) and potassium (K^+^) currents in a concentration-dependent manner with half-maximal effect (EC_50_) values of 193 ± 26 and 93 ± 11 μM and Hill coefficients of 2.52 ± 0.29 and 1.51 ± 0.18 respectively (Szentandrassy et al., 2003). Thymol has been shown to accelerate K^+^-induced contracture in skeletal muscle and inhibit Ca^2+^-binding by the fragmented sarcoplasmic reticulum thus causing the suppression of relaxation (Ebashi, 1965). Thymol (224 μM) was able to invoke the release of Ca^2+^ with an EC_50_ value of 158 ± 16 μM and activate ryanodine receptors in heavy sarcoplasmic reticulum vesicles isolated from skeletal muscle which were loaded with Ca^2+^ (Sarkozi et al., 2007). Thymol (30 μM) was found to increase the depolarization-induced release of Ca^2+^ from the sarcoplasmic reticulum in rodents (Szentesi et al., 2004).

Thymol, at micro concentrations, reduced calcium dependent adenosine triphosphatase (Ca^2+^-ATPase) activity and increased the permeability of Ca^2+^ in the sarcoplasmic membrane and it was found to increase the Ca^2+^ concentrations of neurons or of smooth muscle preparations (Hisayama and Takayanagi, 1986; Kostyuk et al., 1991). Thymol has agonistic effects for the adrenergic receptors (α1, α2, and β) on the circular smooth-muscle strips (SMAs) isolated from stomach and vena portae of guinea pigs. Thymol (10^-4^ M) inhibits spontaneous contractile activity of the SMAs (100%) and diminishes the excitatory effect of acetylcholine chloride to 35%. Thymol via its influence on nerve cell α2-adrenergic receptors showed an analgesic effect (Beer et al., 2007). Thymol (500 μM) was shown to activate the transient receptor potential channel (TRPV3) of the tongue and nasal epithelium (Boudry and Perrier, 2008). In transient receptor potential ankyrin1 (hTRPA1) expressing human embryonic kidney cells (HEK293 cells), thymol (6.25 and 25 μM) activated the response of the membrane potential and increased intracellular Ca^2+^ (Lee et al., 2008). Piglets fed diets supplemented with a combination of thymol (100 and 200 mg/kg) and benzoic acid promoted nutrient digestion and absorption, reduced diarrhea and maintained a favorable micro-environment in the gut (Diao et al., 2015). Thymol (10^-3^ M) lysed dissociated mouse pancreatic acinar cells as evidenced by increased amylase secretion and the secretion of lactate dehydrogenase (LDH) by 315% (Singh, 1980).

A report from Manabe et al. (1987) revealed the biphasic effects of thymol on hypnotic hemolysis. Thymol had a protective effect on erythrocytes at 0.06–1 mM whereas at 2–4 mM it showed a lytic effect on erythrocytes. At a concentration of 1 mM, thymol showed maximum protection for erythrocytes. Thymol (0.2–4 mM) increased the leakage of glutamic oxaloacetate transaminase (GOT) in hepatocytes isolated from male Sprague-Dawley rats. At 33°C, thymol (1 mM) depressed the phase transition temperature and thereby possessed a significant effect on membrane fluidity and it reduced the surface tension from 72 to 53 dye/cm (Manabe et al., 1987). Thymol (0.75–2 mM) triggered the production of superoxide radicals in blood leukocytes in a concentration dependent manner (Suzuki et al., 1987). In guinea pig neutrophils, thymol (1 mM) stimulated superoxide radical production and this was dependent on the initial density of the binding sites and the initial intracellular adenosine triphosphate (ATP) concentrations (Suzuki and Furuta, 1988). Thymol (300 μg/ml) stimulated cytotoxicity whereas treatment with thymol (30–300 μg/ml) dose dependently inhibited the synthesis of deoxyribonucleic acid (DNA), ribonucleic acid (RNA) and protein in cultured mammalian cells (Arai, 1988). Thymol (5%) treatment increased the passive transport of leutinizing hormone-releasing hormone (LHRH) in the porcine epidermis (Bhatia and Singh, 1998). The partition coefficient of thymol was assessed to be 1.65 ± 0.01 × 10^-2^ and at the same concentration it enhanced the permeability of tamoxifen through the porcine epidermis (Gao and Singh, 1998). Thymol (<0.05 mM) suppressed the action potential and reduced membrane resistance and potential in the stomach of guinea pigs at higher concentrations. Thymol (1 mM) inhibited the generation of spikes, hyperpolarized the membrane and resistance in the rectum and ileum (Ito and Kuriyama, 1974).

Thymol (0.002 and 0.00015 mol/L) has been shown to induce nerve blocking action in the phrenic nerve of rats (Seeman et al., 1974). In rabbit white muscle, thymol (0.6 mM) induced accumulation of Ca^+2^ in the sarcoplasmic reticulum (Takishima et al., 1979). Thymol in 0.4% aqueous ethanol has been shown to reduce contractions (ED_50_= 0.86 × 10^-4^ M) in the guinea pig ileum induced by acetylcholine (Van den Broucke and Lemli, 1980). Thymol, a Ca^2+^ antagonist at the concentration of 1 × 10^-4^ M, also reduced the contractions of rat vas deferens induced by 1-noradrenaline via blocking nerve fiber conductions and inhibited the contractions of guinea pig ileum induced by carbachol, histamine and dimethyl phenyl piperazinium (Van den Broucke and Lemli, 1982).

Viana et al. (1981) reported that thymol (2 mg/kg) enhanced the contractions of isolated phrenic-diaphragm and muscles in rats. The same authors revealed that thymol (100 μg) showed potent spasmolytic effects by decreasing the amplitude of peristaltic movements and muscle tone in rabbit duodenum. Thymol (10–300 mg/L) attenuated the force as well as rate of atrial contractions in guinea pigs and at a concentration of 10–300 μg/ml it decreased the aortic contractions isolated from New Zealand white rabbits. Thymol (0.001–0.01%) possessed the ability to relax tension in rabbit intestinal muscles (RIFM 2001, unpublished). Thymol (20 or 40 mg/day) dissolved in olive oil showed clear thyroid activation as detected by oxygen consumption and histological examinations in guinea pigs (Moller, 1939). Thymol (30–300 μg/L) showed a concentration dependent inhibition of DNA, RNA and proteins in V79 cells (Instituto Superiore di Sanita, 1999). Thymol (0.005 M) was shown to possess the ability to convert toxic metals into their non-toxic forms by forming metal complexes or by converting them into their reduced forms by virtue of its antioxidant property (Kishwar et al., 2013).

Thymol (200 mg/kg) attenuated Chang’s disease in male Balb/c mice by reducing parasitemia, trypomastigotes, heart amastigotes and inflammatory infiltrates by its anti-Trypanosomaruzi effect (Juan et al., 2015). Supplementation of thymol rich sources like sage, rosemary extracts and pepper improved the digestibility of feed and the final performance in production (Hernandez et al., 2004). Thymol (2 and 3 g/kg) administration improved various growth parameters such as food conversion ratio, final weight, body growth and composition of tissues (whole body lipids, fibers and proteins) (Ahmadifar et al., 2011). Zheng et al. (2009) reported that thymol has a positive effect on the growth performance of channel catfish (*Ictalurus panctatus*). The presence of thymol might be responsible for the antispasmodic effect of the thyme extract (Engelbertz et al., 2012). Thymol (0–250 μM) showed a weak genotoxic effect in L5178Y/*Tk*^±^ cells as analyzed by the micronucleus (MN) test and mouse lymphoma (MLA) assays (Maisanaba et al., 2015). Thymol (100 μM) blocked voltage-gated sodium channels in stably transfected HEK 293 cells expressing α-subunit of rat brain IIA or hSkM1 sodium channels and this is attributed to its antinoceptive and anesthetic properties (Haeseler et al., 2002).

## Thymol In Cancer Cells

**FIGURE 1:**
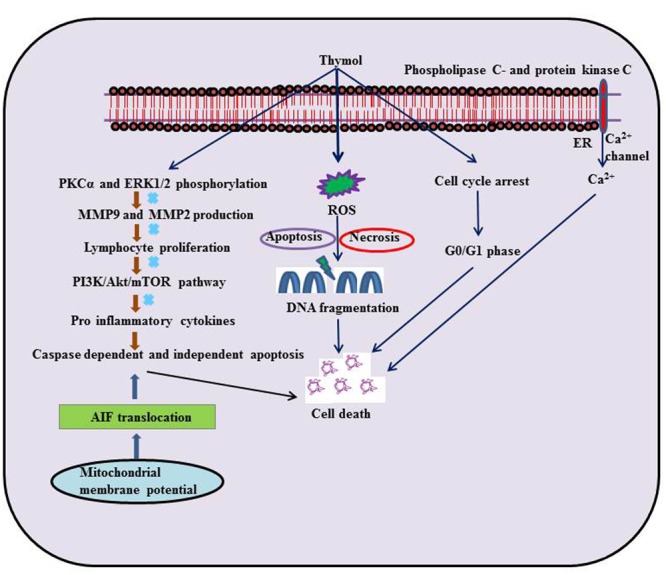
Schematic representation on the effects of thymol in different experimental models of cancer.

Thymol showed anticancer properties in different types of cell lines mimicking human cancer and it demonstrated its potential as a chemopreventive or anticancer agent in various types of cancers. The protective effect of thymol against various types of cancers is represented in **Table 1** and the schema of the protective effects of thymol shown in the studies is represented in **Figure 1**. The major mechanisms of anticancer actions of thymol include induction of apoptosis, anti-proliferation, inhibition of angiogenesis and migration as well as the diminution of umourigenesis by modulating the activity of carcinogen metabolizing enzymes.

**Table 1:** Effects of thymol in different experimental models of cancer.

Dose	Model	Target/End points		Reference
		Increase	Decrease	
**Globlastoma**				
200–600 μM	Human glioblastoma cells	Intracellular Ca^2+^ overload, phospholipase-C and protein kinase-C dependent Ca^2+^ release from endoplasmic reticulum, cell death via apoptosis and necrosis	–	Hsu et al., 2011
**Glioma**				
30 μM	C6 glioma cells		PKCα and ERK1/2 phosphorylation; MMP2 & 9 production	Lee et al., 2016
**Breast cancer**				
0.05–1.25 μM	MCF-7 cells	Cytotoxicity by stimulating cell cycle arrest in G0/G1 phase		Jaafari et al., 2012
LC_50_ = 62.5 μg/mL	MCF-7 cells	Cytotoxicity		Melo et al., 2014
IC_50_= 304.81 μg/ml	MCF-7 cells	Cytotoxicity	Cell viability and proliferation	Khadir et al., 2016
**Leukemia**				
0.05–1.25 μM	K-562 cells	Cytotoxicity by stimulating cell cycle arrest in G0/G1 phase		Horvathova et al., 2007; Jaafari et al., 2012
5–100 μM	HL-60 cells	Cell cycle arrest in G0/G1 phase, DNA fragmentation, Bax protein expression, activation of caspase -9, -8 and -3 & concomitant PARP cleavage, AIF translocation	Bcl2 protein expression	Dutta et al., 2011
0.05–1.25 μM	CEM cells	Cytotoxicity by stimulating cell cycle arrest in G0/G1 phase		Jaafari et al., 2012
30, 50, and 70 μg/ml	HL-60 cells	Cytotoxicity, apoptosis, procaspase-3,8 & 9, PARP-1, cleaved PARP-1, Bax, cytosolic cytochrome-c	MMP, Bcl-2, Bcl-xL, p-110α, Akt, p-Akt, mTOR, p-mTOR, p70S6 K, eIF4E,	Pathania et al., 2013
0–500 μg/ml	THP-1 cells	Cytotoxicity	Proliferation	Miguel et al., 2015
0.005 μg/ml	THP-1 cells		5-LOX activity, TNF-α, IL-8, and IL-1β expressions	Tsai et al., 2011
IC_50_ = 113.51 μM	HL-60 cells	Cytotoxicity, antioxidant activity	Cell viability, cell proliferation	Khadir et al., 2016
IC_50_ = 0.8 μg/ml	P388 cells	Cytotoxicity	–	Hirobe et al., 1998
50 and 200 μg/mL	Peripheral blood lymphocytes		Lymphocyte proliferation	Amirghofran et al., 2011
**Mastocytoma**				
0.05–1.25 μM	P815 cells	Cytotoxicity by stimulating cell cycle arrest in G0/G1		Jaafari et al., 2012
**Osteosarcoma**				
Thymol (400 μM/L)	MG63 cells	Cytotoxicity, ROS, Ca^2+^, Mitochondrial pathway of apoptosis, phospholipase C-dependent Ca^2+^ from ER	Cell viability	Chang et al., 2011
**Hepatocellular carcinoma**				
10–300 μg/ml	Hep G2 cells	Antioxidant capacity	MDA, cytotoxicity	Ozkan and Erdogan, 2011
0.1–0.5 mM	HepG2 cells	Cell viability	Cytotoxicity, DNA damage	Horvathova et al., 2014
IC_50_ 497 and 266 mM	H1299 cells	MDA levels, 8-OHdG, levels, DNA damage, cytotoxicity	Cell viability	Ozkan and Erdogan, 2012
<IC_50_ 497 and 266 mM	H1299 cells	Cell viability	DNA damage, cytotoxicity	Ozkan and Erdogan, 2012
**Cervical cancer**				
30.5–244 ng/ml 1.25, 2, and 5.5 mg/kg	HeLa cells Mice Bone marrow cells	Cytotoxicity	Mitotic index	Reema, 2011
IC_50_ = 134.29 μg/ml	HeLa cells	Cytotoxicity	Cell viability and proliferation	Khadir et al., 2016
**Laryngeal carcinoma**				
0.25–2.2 mM	Hep-2 cells	Necrosis		Stammati et al., 1999
15, 30.5, and 61,122,244) ng/ml	Hep-2 cells	Cytotoxicity		Reema, 2011
**Gastric carcinoma**				
10 0–400 μM	AGS cells	Change in morphology (chromatin condensation, cleavage of DNA, cytoplasm shrinkage, membrane blebbing, and formation of apoptotic bodies); cytotoxicity, intracellular ROS, depolarizing MMP, cytochrome-c release, cleavage of caspases, DNA fragmentation, activation of apaf-1, procaspase 9 recruitment, activation of Bax, PARP, caspase-8 and caspase 7 and 9 cleavage	Cell viability	Kang et al., 2016
**Neuroblastoma**				
400 mg/L	N2a cells		Cell proliferation, total antioxidant capacity	Aydin et al., 2016
19, 25, and 50 mg/L	Primary rat neurons	Cell proliferation, total antioxidant capacity		Aydin et al., 2016
**Other**				
50 and 100 μM	SKOV-3 cells		Genotoxicity, DNA damage	Arab et al., 2015
IC_50_ = 15.6, 150, and 250 μg/mL	SW480, MCF7, JET 3 and Vero cells	Cytotoxicity	Cell viability	Yousefzadi et al., 2012
40–100 mg/kg	Rat bone marrow cells	Structural, numerical and total chromosomal aberration, cytotoxicity	Mitotic index	Azirak and Rencuzogullari, 2008
IC_50_ = 120 ± 15 μM/L LC_50_ = 7.81 μg/mL	B16 murine melanoma cells	Cytotoxicity		He et al., 1997; Melo et al., 2014
IC_50_ = 400 μM, 60.09 μg/mL	B16 murine melanoma cells	Total ROS, morphological changes	Cell viability, relative melanogenesis, relative melanin cell	Satooka and Kubo, 2012
IC_50_ = 20–40 μM	HepG2 and Caco-2 cells		DNA damage, cytotoxicity	Slamenova et al., 2007
250 μM	V79 and Caco-2 cells		Oxidative stress	Cabello et al., 2015
0.24%	*Drosophila melanogaster* larvae		Somatic mutations, URE induced spots	Mezzoug et al., 2007
0.1 mM 0.2 mM	Human lymphocytes Human lymphocytes	Cell viability DNA damage	DNA damage Cell viability	Aydin et al., 2005, Aydin et al., 2005
IC_50_= 0.5 mM	Cultured human fibroblasts	Cytotoxicity, inhibition of DNA	–	Chang et al., 2000
200 μM or 30 μg/mL	A549 cells	SHIP1 and SOCS1 mRNA and protein levels	Levels of interleukin-25, interleukin-33, TLR2, TLR4 expression, induction of miR-155 and miR-21 and completely prevented the induction of miR-146a	Khosravi and Erle, 2016
200 μM or 30 μg/mL	H292 cells	SHIP1 and SOCS1 mRNA and protein levels	Levels of IL-25, IL-33, TLR2, TLR4 expression, induction of miR-155 and miR-21 and miR-146a	Khosravi and Erle, 2016
IC_50_ = 293.53 μM	Caco-2 cells	Cytotoxicity	Cell viability and proliferation	Khadir et al., 2016

### Glioblastoma Cells

In human glioblastoma cells, thymol (200–600 μM) produced a rise in (Ca^2+^)_i_ levels by prompting release of phospholipase C and protein kinase C-dependent Ca^2+^ from the endoplasmic reticulum (ER) and entry of Ca^2+^ via non-store-operated Ca^2+^ channels. Furthermore, the cell death induced by thymol was found to involve apoptosis and necrosis as observed in Annexin V/PI staining (Hsu et al., 2011). Further, thymol (6.0 ± 0.11 mg/g) present in the *Zataria multiflora* extract possessed radio sensitizing effect in human glioblastoma cells (Aghamohammadi et al., 2015).

### Glioma Cells

Thymol (30 μM) treatment in C6 glioma cells was found to reduce fetal bovine serum induced migration. It also diminished matrix metallopeptidase-9 (MMP9) and matrix metallopeptidase-2 (MMP2) production as well as protein kinase Cα (PKCα) and extracellular signal-regulated kinases (ERK1/2) phosphorylation (Lee et al., 2016).

### Breast Cancer Cells

In breast cancer cells (MCF-7 cells), thymol (0.05–1.25 μM) stimulated cytotoxicity by arresting the cell cycle in the G0/G1 phase (Jaafari et al., 2012). Thymol triggered cytotoxicity in MCF-7 breast cancer cell lines with an LC_50_ of 2.5 μg/mL (Melo et al., 2014). In another study, Thymol present in the essential oil of *T. lanceolatus* (IC_50_= 304.81 μg/ml) was shown to induce cytotoxicity and proliferation in MCF-7 cells (Khadir et al., 2016).

### Leukemia Cells

Thymol (0.05–1.25 μM) suppressed oxidant (H_2_O_2_)-induced DNA damage in K-562 cells (Horvathova et al., 2007). The ability of thymol to stop the cell cycle in G0/G1 phase of the K-562 cells seems to be due to its anti-tumor activity (Jaafari et al., 2012). Thymol (5–100 μM) triggers cell death and cell cycle arrest at the sub G0/G1 phase by genomic DNA fragmentation pattern on acute promyelotic leukemia cells (HL-60 cells). Thymol increased the production of ROS and mitochondrial H_2_O_2_ thereby depolarizing mitochondrial membrane potential.

Thymol treatment induced caspase dependent apoptosis by up-regulating Bcl-2 associated X protein (Bax) expression and down-regulating B-cell lymphoma (Bcl-2) expression in a dose-dependent manner. It further augmented the activation of caspase-3, 8, and 9 concomitant to Poly ADP ribose polymerase (PARP) cleavage that is the hallmark of caspase-dependent apoptosis. Furthermore, it also promoted the translocation of apoptosis inducing factor (AIF) from the mitochondria to cytosol and to nucleus, which shows its ability to induce caspase-independent apoptosis. Altogether, the observations indicate that thymol-induced cell death includes both caspase-dependent and caspase-independent pathways (Dutta et al., 2011).

Thymol (0.05–1.25 μM) also induced cytotoxicity mediating cell cycle arrest in the G0/G1 phase of T lymphoblastoid cell line (CEM) (Jaafari et al., 2012). A report from Pathania et al. (2013) has revealed that thymol (30, 50, and 70 μg/ml) suppresses the phosphatidylinositide 3-kinases/Protein kinase B/mechanistic target of rapamycin (PI3K/Akt/mTOR) pathway and induced apoptotic cell death mediating both extrinsic and intrinsic pathways in HL-60 cells. A report from Miguel et al. (2015) has revealed that thymol (0–500 μg/ml) triggered an anti- proliferative effect in human acute monocytic leukemia cells (THP-1 cells).

A report from Khadir et al. (2016) has revealed that thymol present in *Thymus lanceolatus* (IC_50_= 113.51 μM) essential oil triggered cytotoxicity in human leukemia HL-60 cells. Thymol (0.005 μg/ml) present in *Thymus vulgaricus* abrogated the activity of 5-lipoxygenase (5-LOX) and reduced the expression of cytokines *viz.* TNF-α, interleukin-1β (IL-1β) and interleukin-8 (IL-8) in THP-1 cells (Tsai et al., 2011). Thymol (400 mg/kg) showed cytotoxicity toward P388 leukemia cells (IC_50_= 0.8 μg/ml) (Hirobe et al., 1998).

Thymol (50 and 200 μg/mL) inhibited inducible lymphocyte proliferation (62.8 and 89.8%) in a concentration dependent manner as the extracts of *Thymus vulgaris, Thymus daenensis* and *Zataria multiflora* (*Labiatae*) were evaluated for their pharmacological effect on mitogen phytohemagglutinin (PHA)-stimulated peripheral blood lymphocytes using a cell proliferation assay (Amirghofran et al., 2011).

### Mastocytoma Cells

In P815 mastocytoma cell lines, thymol (0.05–1.25 μM) showed the enhancement of cytotoxicity by arresting the cell cycle in G0/G1 phase (Jaafari et al., 2012).

### Osteosarcoma Cells

In human osteosarcoma cells (MG63 cells), thymol (400 μM/L) treatment induced a rise in the levels of (Ca^2+^)_i_ by triggering phospholipase C-dependent Ca^2+^ release from the ER and promoting protein kinase-C sensitive store-operated Ca^2+^ channels mediated entry of Ca^2+^. Thymol also triggered ROS mediated apoptotic cell death via mitochondrial pathways in MG63 cells (Chang et al., 2011).

### Hepatocellular Carcinoma Cells

Thymol was shown to inhibit the proliferation of hepatocellular carcinoma (HCC) in the Bel-7402 cell line as analyzed by human 3-(4,5-dimethylthiazol-2-yl)-2,5-diphenyltetrazolium bromide (MTT) assay and acridine orange (AO)/ethidium bromide (EB) florescent staining (Yin et al., 2010). A report from Ozkan and Erdogan (2011) revealed that thymol (10–300 μg/ml) attenuates cytotoxicity in H_2_O_2_ induced cytotoxicity and membrane damage via inhibiting lipid peroxidation in hepatoma G2 cells (Hep G2 cells). Thymol (0.1–0.5 mM) showed a protective effect for DNA against H_2_O_2_ induced DNA damage in human hepatoma HepG2 cell lines (Horvathova et al., 2014).

Thymol (IC_50_= 497 and 266 mM) was shown to induce DNA damage by increasing the levels of lipid peroxidation products; MDA and 8-hydroxy deoxyguanozine (8-OHdG) in parental and drug resistant human non-small cell lung carcinoma cells (H1299 cell lines). Thymol (<IC_50_= 497 and 266 mM) treatment elicit protection against H_2_O_2_-induced cytotoxicity and showed stabilizing effects on membrane and DNA damage in H1299 cells (Ozkan and Erdogan, 2012). Thymol (25–100 μM) also inhibited acetaminophen-induced cytotoxicity in HepG2 cells as evidenced by improved antioxidant activity and reduction in levels of the pro-inflammatory cytokines such as IL-1β and TNF-α (Palabiyik et al., 2016).

### Cervical Cancer Cells

Cervical cancer is a cancer arising from the cervix due to abnormal cell growth that possesses the ability to invade or spread to other parts of the body. Thymol (30.5–244 ng/ml) induced cytotoxicity by inhibiting the growth of HeLa cells in a concentration-dependent manner. The observed inhibition at the concentration 30.5 ng/ml was 74.06–87.25%. This study has revealed that thymol possesses strong antitumor activities by inducing cytotoxicity and decreasing the mitotic index at higher concentrations in HeLa cell lines (Reema, 2011). Thymol present in the essential oil of *T. lanceolatus* (IC_50_= 134.29 μg/ml) was shown to induce cytotoxicity in HeLa cells (Khadir et al., 2016).

### Laryngeal Carcinoma Cells

In Hep-2 cells derived from human larynx carcinoma, thymol (0.25–2.20 mM) treatment showed concentration-dependent inhibition of neutral red uptake (NRU) and total phenol content (TPC) (IC_50_; NRU-0.71 mM and TPC-0.78 mM). It also exhibited concentration-dependent moderate cytotoxicity by inducing necrotic cell death (Stammati et al., 1999). Thymol (15, 30.5, 61,122 and 244 ng/ml) induced moderate cytotoxicity (51.45%) in Hep-2 cell lines (Reema, 2011).

### Gastric Carcinoma Cells

In human gastric AGS cells, Thymol (100–400 μM) showed a change in cell morphology due to chromatin condensation, cleavage of DNA, cytoplasm shrinkage, and membrane blebbing. The beneficial effects in these cells were attributed to the generation of intracellular ROS, depolarization of mitochondrial membrane potential, apoptosis and impeding cell growth via intrinsic mitochondrial pathway and the activation of pro-apoptotic mitochondrial proteins; caspases, Bax and PARP (Kang et al., 2016).

### Neuroblastoma Cells

Thymol (400 mg/L) decreased cell proliferation in cultured neuroblastoma cells (N2a cells) whereas thymol (19, 25, and 50 mg/L) increased the total antioxidant capacity in rat neurons but not in N2a cells. This report clearly revealed that thymol is a potent anticancer and antiproliferative agent (Aydin et al., 2016).

### Other Studies

Thymol (50 and 100 μM) has been reported to inhibit bleomycin induced genotoxicity in human lymphocytes by its chemoprotective effect. It was also shown that thymol pretreatment in bleomycin treated human ovarian carcinoma cells (SKOV-3 cells) neither enhanced cell neither death nor cell protective effects but it prevented bleomycin induced DNA damage in normal cells. This study recommended the combination of thymol with various chemotherapeutic agents to minimize its toxicity on normal cells and to improve the effectiveness of cancer treatment (Arab et al., 2015).

A report from Yousefzadi et al. (2012) has revealed that thymol (40.2%) present in the essential oil of *S. sahendica* (IC_50_= 15.6, 15.6, 125, and 250 μg/ml) significantly reduced cell viability of human colon adenocarcinoma cells (SW480 cells), MCF7, JET3 and monkey kidney cells (Vero cells). Thymol (40–100 mg/kg) induced structural, numerical and total chromosomal aberrations (CA) in rat bone marrow cells and it also has cytotoxic effect in rat bone marrow cells by decreasing the mitotic index (Azirak and Rencuzogullari, 2008). Thymol (0.4 mM) exerted no appreciable effect against mutagenic and carcinogenic heterocyclic amines (HCAs) (Oguri et al., 1998). Thymol (IC_50_= 120 ± 15 μM/L) displayed cytotoxicity on murine B16 melanomas *in vitro* and *in vivo* by its potent anti-tumor effect (He et al., 1997). Thymol (LD_50_= 7.81 μg/mL) present in the *L. gracilis* essential oil was shown to induce cytotoxicity in B16 murine melanoma cell line (Melo et al., 2014).

Thymol triggered cytotoxicity with an IC_50_ value of 400 μM (60.09 μg/mL) along with oxidative stress in B16 melanoma cells. Thymol generates a phenoxy radical intermediate by its potent antioxidant effect followed by the production of ROS and quinine oxide derivatives. The toxicity of thymol at higher doses is due to the formation of antioxidant-related free radicals (Satooka and Kubo, 2012). Thymol (IC_50_= 20–40 μM) showed protective effect against H_2_O_2_ induced DNA double strand breaks in HepG2, human colonic cells (Caco-2 cells) and hamster lung cells (V79 cells) (Slamenova et al., 2007). Thymol (0.24%) present in the essential oil of *Origanum compactum* showed a strong inhibitory effect on indirect-acting mutagen in urethane (URE) induced mutagenicity in *Drosophila melanogaster* as investigated by the somatic mutation and recombination test (SMART test). Thymol suppressed the mutations by 43% (Mezzoug et al., 2007). Thymol (0.1 mM) significantly decreased DNA double strand breaks in 2-amino-3-methylimidazo(4,5-f)-quinoline (IQ) and mitomycin C (MMC) induced DNA damage in human lymphocytes and at higher concentrations of about 0.2 mM, thymol itself induced DNA damage in lymphocytes (Aydin et al., 2005). In the SOS-chromotest and the DNA-repair test the genotoxic potential of thymol was found to be very weak (Stammati et al., 1999).

Thymol (IC_50_= 0.5 mM) induced cytotoxicity by inhibiting DNA in a concentration dependent manner. However, thymol did not cause DNA single strand breaks in cultured human pulp fibroblasts (Chang et al., 2000). Combined treatment with carvacrol/thymol (200 μM, equal to 30 μg/mL) suppressed chitin induced alterations in human lung carcinoma cells (A549 cells) and human lung mucoepidermoid carcinoma cells (H292 cells) (Khosravi and Erle, 2016). Thymol (IC_50_= 293.53 μM) present in the *T. lanceolatus* extract was shown to induce cytotoxicity in Caco-2 cells (Khadir et al., 2016).

## Thymol In Cardiometabolic Diseases

**FIGURE 2:**
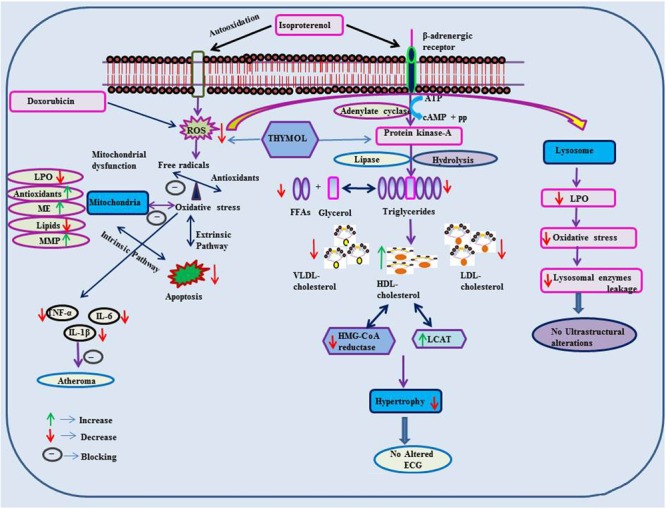
Effect of thymol on ISO and doxorubicin induced cardiotoxicity.

**FIGURE 3:**
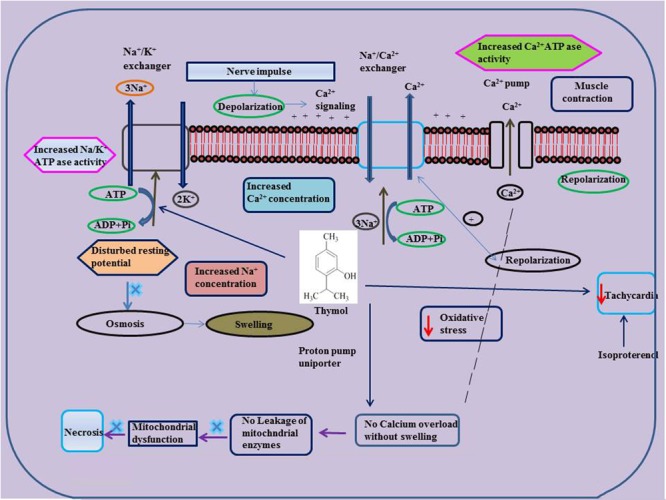
Effect of thymol on ISO induced altered ionic homeostasis and mitochondrial dysfunction.

The protective effects of thymol in various cardiovascular related disorders such as MI, hyperlipidemia and several others are represented in **Tables 2, 3** and the schema of the protective effects of thymol shown in the studies is represented in **Figures 2, 3**.

**Table 2:** Effect of thymol in different animal models of cardiovascular diseases.

Dose	Model	Target/End points		Reference
		Increase	Decrease	
**Myocardial infarction**				
7.5 mg/kg and 50 μM	ISO (100 mg/kg)-induced myocardial necrosis in Male albino Wistar rats	Vitamin-C, vitamin-E, GSH, reducing power	Serum CK-MB, plasma TBARS, LOOH and CDs	Nagoor Meeran and Prince, 2012
7.5 mg/kg and 50 μM	ISO (100 mg/kg)-induced myocardial necrosis in Male albino Wistar rats	Serum HDL-C, HMG-CoA-reductase, LCAT, myocardial gene expression of Bcl-2, DPPH radical scavenging	Serum cardiac troponin-T and I, ST segment elevation, tachycardia, heart weight, left ventricular hypertrophy, serum and heart total cholesterol, TGs, FFAs, LDL-C, VLDL- C, atherogenic index, myocardial Bax gene	Nagoor Meeran et al., 2015b
7.5 mg/kg	ISO (100 mg/kg)-induced myocardial necrosis in Male albino Wistar rats	–	hsCRP, lysosomal TBARS, serum and heart β-glucuronidase, β-galactosidase, cathepsin-B and cathepsin-D, myocardial TNF-α, IL-6 and IL-1β, lysosomal destabilization	Nagoor Meeran et al., 2015b
7.5 mg/kg	ISO (100 mg/kg)-induced myocardial necrosis in Male albino Wistar rats	SOD, catalase, Na^+^/K^+^-ATPase, potassium ion	Serum LDH, troponin-T, heart TBARS, LOOH, Ca^2+^-ATPase, Mg^2+^-ATPase, Ca^2+^ and Na^+^, myocardial infarct size	Nagoor Meeran et al., 2015a
7.5 mg/kg and 50 μM	ISO (100 mg/kg)-induced myocardial necrosis in Male albino Wistar rats	Mitochondrial complex enzymes and cytochrome-C-oxidase, ATP, scavenging of hydroxyl radicals	Serum CK, LDH, mitochondrial TBARS, LOOH, cholesterol, TGs, FFAs, phospholipids, Ca^2+^ overload	Nagoor Meeran et al., 2016b
7.5 mg/kg and 50 μM	ISO (100 mg/kg)-induced myocardial necrosis in rats	Myocardial CK, H_2_O_2_ radical scavenging	Plasma uric acid, protein, hexose, hexosamine, fucose and sialic acid	Nagoor Meeran et al., 2014
7.5 mg/kg	ISO (100 mg/kg)-induced myocardial necrosis in rats	Heart LOOH, GPx, GSH, vitamin-C, vitamin-E and expression of BcL-xL	Serum CK, gene expressions of caspase-8, caspase-9 and Fas	Nagoor Meeran et al., 2016b
**Drug induced cardiotoxicity**				
20 mg/kg	Doxorubicin (10 mg/kg)-induced male Swiss Albino rats	SOD, catalase, GSH in the heart	Serum LDH, CK, CK-MB, AST, TNF-α, MDA, cardiac troponins	El-Sayed et al., 2016
10–100 mg/kg	Cardiotoxin (25 μM)-induced toxicity	Regeneration of skeletal muscle	Inflammation of skeletal muscle, collagen area	Cardoso et al., 2016
**Atheroscelerosis**				
3 and 6 mg/kg	New Zealand white male rabbits	Antioxidant activity, HDL-C	TGs, total cholesterol, LDL-C, MDA, hsCRP, intimal thickening of aorta, messenger RNA expression of IL-1 β, IL-6, TNF-α, TNF-β, VCAM-1, MCP-1, and MMP-9	Yu et al., 2016
5–25 μg/mL	oxLDL-stimulated THP-1-macrophages	IL-10 expression	TNF-α, IL-1β, and IL-6 expressions, translocation of NF-κB into the nucleous	Ocana-Fuentes et al., 2010; Ocana and Reglero, 2012
1.25–10 μM	Human aortic endothelial cells	Antioxidant activity	CDs, LDL-oxidation	Pearson et al., 1997
**Hypertension**				
300, 400, and 1000 μM	Rat isolated aorta	Ca^2+^ release	PHE induced Endothelial ring contractions CaCl_2_ induced contractions in Ca^2+^ free medium	Peixoto-Neves et al., 2010
1–10 mg/kg	Male or female Wistar rats	–	Systolic, diastolic, and mean arterial pressure, heart rate	Aftab et al., 1995
10–300 μg/ml (IC_50_ = 100 μg/ml)	Guinea pig atria	Vasorelaxation	Force and rateg of atrial contractions, K^+^ induced contractions	RIFM 2001, unpublished
10–300 μg/ml	Rabbit aorta	Vasorelaxation		RIFM 2001, unpublished
1, 3, and 10 mg/kg	Wistar rats	–	Blood pressure and heart rate	RIFM 2001, unpublished
5 mg/kg	Rabbits		Blood pressure	RIFM 2001, unpublished
**Arrythmias**				
10, 100, and 250 μM	Canine ventricular cardiomyocytes	*I*_Ca_ inactivation	K^+^ and Ca^2+^ currents, action potential, V_max_	Magyar et al., 2002

**Table 3:** Effect of thymol in different experimental models of metabolic disorders and nephrotoxicity.

Dose	Model	Target/End points		Reference
		Increase	Decrease	
**Diabetes Mellitus**				
40 mg/kg	High fat diet induced C57BL/6J mice	Adiponectin, LCAT, LPL, HDL-C, CPT, ME, PAP	Body weight, HOMA-IR, HbA1c, insulin, glucose, leptin, HMG-CoA reductase, plasma and hepatic lipid profile, fatty acid β-oxidation, activities of G6PD and FAS	Saravanan and Pari, 2015
40 mg/kg	High fat diet induced C57BL/6J mice	Serum protein,, SOD, catalase, GPx, GRx, GST, GSH, vitamin-C, vitamin-E in erythrocyte and kidney	Blood glucose, insulin, BUN, creatinine, TBARS, LOOH, erythrocytes and kidney, total cholesterol, TGs, FFAs, PLs in kidney,, gene expressions of SREBP-1c, TGF-β1, VEGF, lipid accumulation	Saravanan and Pari, 2016
0.5–2.0 mg/ml	AAPH induced diabetic erythrocytes	Free radical scavenging	Lipid peroxidation, RBC hemolysis	Aman et al., 2013
**Obesity**				
30 mg/kg	HFD-induced murine model	HDL-C levels, SOD and catalase in serum	Body weight, food intake, serum and hepatic function parameters and lipid profile	Haque et al., 2014
20 μM	3T3-L1 white adipocytes	Expressions of signaling molecules of glucose homeostasis and lipid metabolism	Cytotoxicity, LPL expression, TG accumulation	Choi et al., 2016
**Nephrotoxicity**				
20 mg/kg	Cisplatin induced male adult Swiss albino rats	Antioxidants in kidney	Decrease creatinine and BUN, TNF-α, caspase-3 and MDA	El-Sayed et al., 2014
200–500 μM	MDCK cells	Ca^2+^, apoptosis, ROS	Cell viability	Chang et al., 2014
50 and 150 mg/kg	Cisplatin induced Swiss albino mice	Uptake of 99mTc-DMSA (dimer captosuccinic acid)	Tubular necrosis, degeneration, epithelial vacuolization, swelling	Hosseinimehr et al., 2015

### Myocardial Infarction

Thymol (7.5 mg/kg) was shown to inhibit the occurrence of oxidative stress in rats challenged with ISO, an agent which commonly induces myocardial necrosis. The benficial effects were attributed to decreased levels of lipid peroxidation products such as thiobarbituric acid reactive substances (TBARS), lipid hydroperoxides (LOOH) and conjugated dienes (CDs) in plasma. Further, it also normalized non-enzymatic antioxidants such as vitamin-C, vitamin-E and GSH in the plasma due to its potent antioxidant action (Nagoor Meeran and Prince, 2012). Furthermore, thymol attenuates altered lipid metabolism [decreased the levels/concentrations of serum and heart lipids such as total cholesterol, triglycerides (TGs) and free fatty acids (FFAs)], reinstating the normal levels of lipoproteins (increased HDL-C with decreased LDL-C and VLDL-C levels in the circulation) in ISO-induced myocardial infarcted rats. Thymol was shown to attenuate the alterations in the activities of lipid marker enzymes such as 3-hydroxy-3-methyl-glutaryl-coenzyme A reductase (HMG-CoA reductase) and lecithin–cholesterol acyltransferase (LCAT) in the liver, inhibiting tachycardia (increased heart rate), decreasing atherogenic index, and the levels of serum cardiac troponins, altered electrocardiographic patterns (ST segment elevation), cardiac hypertrophy (decreased heart weight and left ventricular weight/body weight) and apoptosis (increased expression of myocardial Bcl-2 gene and decreased expression of Bax-gene in ISO-induced myocardial infracted rats) (Nagoor Meeran et al., 2015b). Also, thymol has been shown to attenuate inflammation of the myocardium by inhibiting the release of lysosomal enzymes (β-glucuronidase, β-galactosidase, cathepsin-B and cathepsin-D) from the heart to the circulation by decreasing the levels of lysosomal TBARS, release of inflammatory marker such as high sensitive C-reactive protein (hsCRP) and down regulating the myocardial expressions of pro-inflammatory cytokines such as TNF-α, interleukin-6 (IL-6) and IL-1β genes in ISO-induced myocardial infracted rats. The transmission electron microscopic findings revealed preservation of lysosomal architecture and histopathological salvage in concurrence with the biochemical observations (Nagoor Meeran et al., 2015b).

Oral administration of thymol abrogates myocardial membrane destabilization by inhibiting myocardial oxidative stress (decreased concentrations of lipid peroxidations products in heart and improved activities of antioxidant enzymes), reduced leakage of the cardiac marker enzyme LDH into the circulation, decreasing the activity of Ca^2+^ ATPase and increasing the activity of sodium/potassium dependent adenosine triphosphatase (Na^+^/K^+^ ATPase) in ISO-induced infarcted rats. Furthermore, thymol also increased K^+^ concentrations and enhanced sodium (Na^+^) and Ca^2+^ concentrations in the heart. Also, thymol significantly diminished the myocardial infarct size as analyzed by 2,3,5-triphenyl tetrazolium chloride (TTC) assay due its potent membrane stabilizing property (Nagoor Meeran et al., 2015c). Thymol was shown to inhibit mitochondrial dysfunction in ISO-induced myocardial necrosis in rats. Pre and co-treatment with thymol showed decreased heart mitochondrial lipid peroxidation products (TBARS and LOOH), lipids (cholesterol, TGs, FFAs and phospholipids (PLs), Ca^2+^ and significant increase in the activities of heart mitochondrial antioxidants (SOD, catalase, GPx, GSH) and mitochondrial marker enzymes such as isocitrate dehydrogenase (ICDH), malate dehydrogenase (MDH), α-ketoglutarate dehydrogenase (α-KGDH), reduced nicotinamide adenine dinucleotide dehydrogenase (NADH dehydrogenase) and cytochrome-C-oxidase) in ISO-induced MI in rats. It also enhanced the ATP levels and diminshed the mitochondrial swelling. Transmission electron microscopic study on heart mitochondria confirmed the biochemical findings of the study. This study revealed the ability of thymol in protecting the heart mitochondria against ISO induced oxidative stress in rats (Nagoor Meeran et al., 2016b).

Thymol has been shown to decrease the levels of plasma uric acid and glycoprotein components viz. hexose, hexosamine, fucose and sialic acid in ISO-induced rats due to its potent antioxidant property (Nagoor Meeran et al., 2014). Thymol was shown to inhibit apoptosis by decreasing oxidative stress in ISO-induced myocardial infracted rats. Thymol treatment decreased the concentrations of lipid peroxidation products and increased the status of antioxidants in the myocardium such as GPx, GSH, vitamin-C and vitamin-E. It also decreased the myocardial gene expressions of caspase-8, 9 and Fas genes and increased the expressions of B-cell lymphoma extra-large (BcL-xL) gene. Histopathological and the *in vitro* ferric reducing antioxidant power (FRAP) assay confirmed the biochemical observations. This study revealed the protective effect of thymol against apoptotic cell death in the heart by attenuating oxidative stress (Nagoor Meeran et al., 2016a). In all these studies, thymol pre- and co-treatment in rats appear devoid of any deleterious effects which is suggestive of its safety. These preclinical studies recommended the clinical trials to reveal the exact dosage of thymol against MI in humans.

### Doxorubicin Induced Cardiotoxicity

Thymol has been shown to abrogate oxidative stress, inflammation and apoptosis in doxorubicin induced cardiotoxicity in rats. Thymol (20 mg/kg), in pre- and co-treated rats, was shown to decrease the levels of serum LDH, aspartate transaminase (AST), creatine kinase (CPK), creatine kinase-MB (CK-MB), cardiac troponin-I and TNF-α with decreased concentrations of caspase-3 and MDA in the heart. The activities of antioxidants such SOD, catalase and GSH were shown to increase in thymol pre- and co-treated doxorubicin-induced cardiotoxic rats. This study has shown that the combined treatment of thymol and carvacrol revealed a much better effect than the treatment with thymol and carvacrol alone in doxorubicin-induced cardiotoxic rats. But, thymol possesses a more superior effect than its isomer carvacrol in the same model and the actions are attributed to the antioxidant, anti-inflammatory, and antiapoptotic activity of thymol (El-Sayed et al., 2016). A report from Cardoso et al. (2016) has revealed that thymol (10–100 mg/kg) attenuates inflammation and recovers skeletal muscle from cardiotoxicity in mice.

### Atheroscelerosis

A report from Yu et al. (2016) showed that thymol attenuates oxidative stress, aortic intimal thickening, and inflammation by regulating gene expression in hyperlipidemic rabbits. Thymol (3 and 6 mg/kg) supplementation has been shown to decrease the levels of TGs, total cholesterol, LDL-C, MDA, high sensitive C-reactive protein, intimal thickening of aorta with increased levels of HDL-C and total antioxidant capacity in hyperlipidemic rabbits induced by giving a high fat diet. Furthermore, thymol (3 and 6 mg/kg) was shown to decrease the mRNA expressions of IL-1β, IL-6, TNF-α, TNF-β, Vascular cell adhesion protein 1 (VCAM-1), monocyte chemo attractant protein-1 (MCP-1) and MMP-9 in hyperlipidemic rabbits. Thymol (121.4 μM) effectively scavenged DPPH and 2,2′-azino-bis(3-ethylbenzothiazoline-6-sulphonic acid (ABTS) radicals which revealed its potent antioxidant and free radical scavenging properties. Finally, thymol administration lowered serum lipids and attenuated oxidative stress followed by an inflammatory response in hyperlipidemic rabbits. This study recommended further studies to reveal the mechanism of action of thymol on endothelial dysfunction and smooth muscle cell migration (Yu et al., 2016). Thymol (5–25 μg/mL) administration showed decreased expressions of pro-inflammatory cytokines (TNF-α, IL-1β and IL-6) with increased expression of IL-10 that inhibited translocation of NF-κB into the nucleus in the oxidative-LDL induced THP-1 macrophages, a cellular model of inflammation/atherogenesis (Ocana-Fuentes et al., 2010; Ocana and Reglero, 2012). In human aortic endothelial cells, thymol (1.25–10 μM) produced a concentration dependent inhibition of oxidation of LDL-C (Pearson et al., 1997).

### Hypertension

Thymol has been shown to exhibit vasorelaxant activities in the isolated rat aorta. Thymol showed relaxation on aortic ring preparations in a concentration dependent manner using potassium chloride (KCl) or using phenylephrine (PHE) (IC_50_ value of 64.40 ± 4.41 and 78.80 ± 11.91 μM) and (PHE, 0.1 μM) (IC_50_ value of 106.40 ± 11.37 and 145.40 ± 6.07 μM). In isolated rat aorta, endothelium-independent relaxation induced by thymol occurs via release of Ca^2+^ from the sarcoplasmic reticulum diminishing the sensitivity of contractile elements to Ca^2+^ and preventing the influx of Ca^2+^ across the membrane (Peixoto-Neves et al., 2010). Thymol (1–10 mg/kg) showed a dose dependent decline in blood pressure and heart rate in rats. Also, it decreased the force and rate of atrial contractions in spontaneously beating atria (Aftab et al., 1995). Thymol (10–300 μg/ml) (IC_50_= 100 μg/ml, 0.1 mM) dose dependently triggered the relaxation of potassium and norepinephrine induced contractions in the rabbit aorta. Thymol by virtue of its Ca^2+^ channel blocking effect expressed its hypotensive and bradycardiac effects in various animal studies (Aftab et al., 1995). Thymol (1, 3, and 10 mg/kg) administration decreased the blood pressure and heart rate of Wistar rats whereas thymol (5 mg/kg) attenuated blood pressure in rabbits (RIFM, 2001, unpublished).

### Cardiac Arrythmias

Thymol (10 and 100 μM) induced cardiac arrhythmias via concentration-dependent inhibition of K^+^ and Ca^2+^ currents in canine ventricular cardiomyocytes using microelectrode and patch clamp techniques. Thymol (10 μM) ablated the action potential notch whereas thymol (100 μM) decreased the duration of the action potential, reduced maximum velocity (V_max_) and the depression of the plateau. These results are found in line with the activity of thymol in ventricular myocytes isolated from healthy human hearts (Magyar et al., 2002). Thymol (10–1000 μM) inhibits the effect of L-type Ca^2+^ currents in human and canine ventricular myocytes using the ‘patch clamp technique’ in the ‘whole-cell’ configuration on the inactivation of the channel machinery (Magyar et al., 2004). Thymol triggers negative inotropic actions in canine and guinea pig preparations in a concentration-dependent manner. At lower concentrations, thymol reduced intracellular Ca^2+^ transients without altering the contractile function whereas Ca^2+^ transients and at higher concentrations suppressed contractions in guinea pig hearts. Thymol reduced the activity of Ca^2+^ pump by inducing rapid release of Ca^2+^ in canine sarcoplasmic reticular vesicles (Szentandrassy et al., 2004).

## Thymol In Metabolic Disorders

**FIGURE 4:**
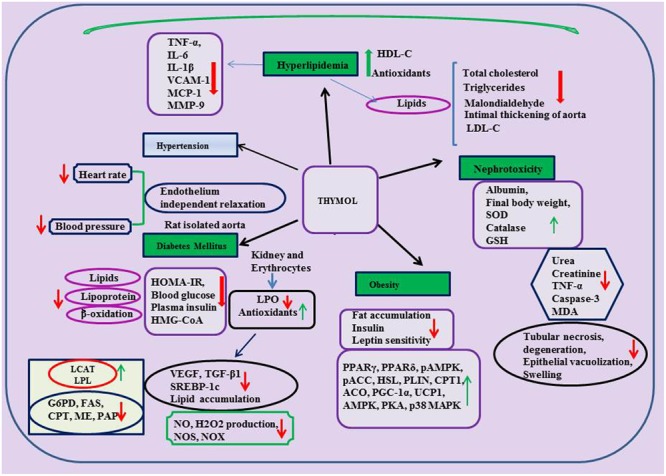
Effect of thymol on metabolic and kidney disorders.

The protective effects of thymol in metabolic disorders such as diabetes mellitus and obesity are represented in **Table 3** and the schema of the protective effects of thymol shown in the studies are depicted in **Figure 4**.

### Diabetes Mellitus

Thymol was shown to protect against various metabolic disorders. A report from Saravanan and Pari (2015) has revealed the anti-hyperglycemic and hyperlipidemic activity of thymol in high fat diet induced type-2 diabetes in C57BL/6J mice. Thymol (40 mg/kg) administration was shown to reduce final body weight, HOMA of insulin resistance (HOMA-IR), glycosylated hemoglobin (HbA1c), plasma insulin and blood glucose in high fat diet induced type-2 diabetes in C57BL/6J mice. Thymol suppressed plasma and hepatic levels of total cholesterol, TGs, FFAs, PLs, LDL-C and significantly increased the levels of HDL-C in high fat diet induced mice. Furthermore, thymol treatment increased the levels of adiponectin and decreased the levels of leptin in high fat diet (HFD) mice. Also, thymol inhibited alterations in the activities of lipid metabolizing enzymes (significant increase in the activities of LCAT, lipoprotein lipase (LPL) and decrease in the activities of HMG-CoA reductase in HFD mice). Thymol treatment reduced the levels of fatty acid β-oxidation and the activities of glucose 6-phosphate dehydrogenase (G6PD), fatty acid synthase (FAS) along with increased activities of carnitine palmitoyl transferase (CPT), malic enzyme (ME) and phosphatidate phosphohydrolase (PAP) in HFD mice (Saravanan and Pari, 2015).

Another study reported by the same group, has revealed that thymol abrogated diabetic nephropathy in HFD-induced diabetes in C57BL/6J mice (Saravanan and Pari, 2016). Thymol (40 mg/kg) treatment for a period of 5 weeks reduced blood glucose level and improves the parameters of renal function. Thymol treatment also suppressed the activation of vascular endothelial growth factor (VEGF) and transforming growth factor-β1 (TGF-β1) and down regulated expression of sterol regulatory element binding protein-1c (SREBP-1c) and reduced lipid accumulation in the kidneys. Extracellular mesangial matrix expansion and glomerulosclerosis were suppressed also by thymol in HFD induced mice as evidenced in histological studies and it also enhanced antioxidant status and inhibited lipid peroxidation in erythrocytes and kidneys. Thymol (0.5–2.0 mg/ml) has been shown to protect red blood cells (RBCs) from 2,2-azo-bis(2-amidinopropane) dihydrochloride (AAPH) induced hemolysis in diabetic patients due to its potent antioxidant and free radical scavenging effect (Aman et al., 2013). According to the report of Kavoosi and Teixeira da Silva (2012), thymol reduced NO, H_2_O_2_ production along with NOS, NADH-oxidase (NOX) activities in human monocytes cultured in the presence of 20 mM glucose. Thymol present in the methanolic extract of *Thymus quinquecostatus* showed inhibitory effect on the enzymes α-amylase and α-glucosidase responsible for breakdown of carbohydrates and further intestinal absorption (IC_50_= 4.39 ± 0.22 μg/ml) (Hyun et al., 2014). The findings demonstrate that thymol has promising potential in the treatment of hyperglycemia and associated complication.

### Obesity

Obesity is defined as excessive adiposity and is one of the major health and socioeconomic burdens which leads to a number of chronic diseases such as coronary heart disease (Sedova et al., 2004), diabetes (Lazar, 2005; Sanchez-Castillo et al., 2005), hyperlipidemia (Jeusette et al., 2005) and various cancers (Stunkard and Allison, 2003a,b; Stunkard et al., 2003). Thymol (30 mg/kg) was shown to inhibit the accumulation of visceral fats, enhance insulin and leptin sensitivity and improve lipid lowering action as well as augment antioxidant status in HFD-induced obesity in murine models (Haque et al., 2014). Thymol (20 μM) has been shown to promote the biogenesis of mitochondria and increase the expression of brown fat-specific markers along with improved expressions of peroxisome proliferator activated receptor-γ (PPARγ), peroxisome proliferator activated receptor-δ (PPARδ), phospho AMP-activated protein kinase (pAMPK), pampk; Phospho acyl-CoA carboxylase (pACC), hormone-sensitive lipase (HSL), perilipin (PLIN), carnitine palmitoyltransferase-1 (CPT1), acyl-coenzyme A oxidase-1 (ACO), peroxisome proliferator-activated receptor gamma co-activator 1-alpha (PGC-1α), and uncoupling protein 1 (UCP1) in the browning of white adipocytes (3T3-L1 white adipocytes) which play an important role in glucose homeostasis and lipid metabolism. Altogether, the findings reveal that thymol has the potential to regulate oxidation of fatty acids, lipolysis augmentation, lipolysis reduction and thermogenesis. Thymol possesses the ability to activate the β3-adrenergic receptor along with AMPK-activated protein kinase (AMPK), protein kinase-A (PKA), and p38 mitogen-activated protein kinase (p38 MAPK) pathways and it could be the reason for its ability to trigger UCP1 expression in other brown fat-specific markers (Choi et al., 2016).

## Thymol In Renal Diseases

The protective effects of thymol in renal diseases are represented in **Table 3** and the schema of the protective effects of thymol shown in the studies are represented in **Figure 4**. Thymol (20 mg/kg) was shown to inhibit cisplatin-induced renal injury by attenuating oxidative stress, inflammation and apoptosis in male adult Swiss Albino rats (El-Sayed et al., 2014). Thymol (200–500 μM) induced Ca^2+^ release from the ER which facilitated the entry of Ca^2+^ via store-operated Ca^2+^ entry in Madin-Darby canine kidney (MDCK) renal tubular cells. Thymol triggers cell death by promoting apoptosis mediated by ROS in MDCK renal tubular cells (Chang et al., 2014). Thymol’s (50 and 150 mg/kg) beneficial effect on cisplatin-induced renal injury in mice was also demonstrated by quantitative renal dimer captosuccinic acid (^99m^Tc-DMSA) uptake concomitant to potent antioxidant and anti-inflammatory properties. ^99m^Tc-DMSA uptake per gram tissue of kidneys in %ID/g was 65.02 ± 32.21 and 88.46 ± 20.46 in the thymol (50 and 150 mg/kg) treated mice induced with cisplatin. Furthermore, Thymol administration increased the level of %ID/g (Hosseinimehr et al., 2015).

## Thymol In Gastrointestinal Disorders

The protective effects of thymol in gastrointestinal disorders are represented in **Table 4**. Nowadays, the prevalence of inflammatory diseases in the intestines are rising as a serious problem in humans. The increased expressions of pro-inflammatory cytokines such as interleukin-1 (IL-1), IL-6, IL-8, TNF-α, IL-12, and IFNγ were reported in the inflamed intestinal mucosa of both animal and humans (Rogler and Andus, 1998; Wirtz and Neurath, 2000; Bertevello et al., 2005; Raddatz et al., 2005; Bukovska et al., 2007). Thymol present in thyme and oregano oils (0.05–0.4%) was shown to inhibit 2,4,6-trinitrobenzenesulfonic acid-induced colitis by decreasing the mRNA expressions of pro inflammatory cytokines (IL-1β, IL-6, granulocyte-macrophage colony-stimulating factor (GM-CSF) and TNF-α) and protein expressions of IL-1β and IL-6 in mice (Bukovska et al., 2007). A report from Platel and Srinivasan (2004) demonstrated the ability of thymol to prompt secretion of salivary amylase in humans and of bile acids, gastric and pancreatic enzymes such as lipase, amylase and proteases and intestinal mucosa in rats. Thymol administration increased the activities of pancreatic amylase, maltase and trypsin in broiler chickens (Jang et al., 2007).

**Table 4:** Effect of thymol in different experimental models of gastrointestinal diseases.

Dose	Model	Target/End points		Reference
		Increase	Decrease	
**Colitis**				
0.05–0.4%	Male 7-week-old Balb/c mice		Epithelial necrosis, gland destruction, inflammatory cell infiltration, mRNA expressions of IL-1β, IL-6, GM-CSF, and TNFα, protein expressions of IL-1β and IL-6	Bukovska et al., 2007
**Ulcer**				
100 mg/kg	Adult male Albino Wistar rats	Mucus production, prostaglandins, ATP-sensitive K^+^ channels	Total lesion, mucus damage, epithelial cell loss, oedema, ulcer index	Ribeiro et al., 2016
100 μM 1.3 mg/kg	Female wistar rats C57BL/6 mice	Mucociliary clearnance	K^+^ and Ba^2+^ tracheal contraction	Begrow et al., 2010
10 mg/kg	Adult male Albino Wistar rats	SOD, GSH	Mucosal damage, folding of the internal elastic lamina of small arteries, neutrophil infiltration, lipid peroxidation, MPO, MMP-2	Chauhan and Kang, 2015
**Other**				
10–100 μM	Large white, Landrace, Pietrain piglets	Short circuit current	Cl^-^ and HCO3^-^ secretion	Boudry and Perrier, 2008
0.015%	Adult male albino rats		Percentage of contraction	Hejazian et al., 2014
0–2 mM	Myosin from fast skeletal muscles of Japanese white rabbits	S1 ATPase, acto-S1ATPase myosin cross bridges	Isometric force, velocity of shortening, isometric force	Tamura and Iwamoto, 2004
50 mg/kg	Male weaned pigs	Pancreatic digestion related genes including somatostatin receptor 2 and calpain 9, serotonin receptor 2A	Cation channel activity and gated channel activity gene transient receptor potential cation channels, ryanodine receptors 2 and 3, and some voltage-dependent Ca^2+^ channel genes, potassium voltage-gated channel subfamily A member 1 and 2 some large-conductance Ca^2+^ -activated potassium channel genes	Colombo et al., 2014
**Hepatotoxicity**				
30 mg/kg + hydrocortisone (5 mg/kg)	Male albino Wistar rats	Total protein, albumin, TAC, liver GSH	Serum AST, ALT, TOC, liver TBARS, TNF-α in the serum and liver	Aboelwafa and Yousef, 2015
300 mg/kg + CCl_4_ (5 ml/kg)	Male Swiss albino mice	SOD, GPx	MDA, serum ALT, fatty changes, necrosis and lymphocyte infiltration	Al-Malki, 2010
300 mg/kg + CCl_4_ (20 μl/kg)	Male Swiss albino mice	–	Serum ALT, MDA and TBARS in the liver, hepatocellular necrosis	Alam et al., 1999
150 mg/kg + paracetamol (640 mg/kg)	Male Swiss albino mice	Hepatic ALP, AST and ALT	Mortality, serum ALP, AST and ALT	Janbaz et al., 2003
0–50 μg/ml	Chang liver cells	MMP, GSH, Bcl_2_	Cytotoxicity, apoptosis, ROS, MDA and Bax	Kim et al., 2014
125 mg/kg + CCl_4_	CCl_4_ induced female Swiss OFFI mice	Serum glutamic pyruvate transaminase	Hepatic MDA	Jimenez et al., 1993
1 and 9.73 ml/kg	Albino Wistar rats of both sexes	Total bilirubin, direct bilirubin, AST, ALT, urea, creatinine, catalase, GPx, GRx	Cholesterol, TGs, lipid peroxidation, xanthine oxidase	Raskovic et al., 2015
25–100 μM	HepG2 cells	Cell viability, SOD and GSH	MDA, ALT, LDH, gene expressions of TNF-α and IL-1β	Palabiyik et al., 2016
**Other**				
50–200 mg/kg	Swiss albino mice	Phase I and II enzymes		Sasaki et al., 2005

Thymol (100 mg/kg) has been shown to attenuate acute and chronic ulcers induced by various agents such as ethanol, indomethacin and acetic acid by attenuating the inflammatory process, i.e., infiltration of inflammatory cells and edema. This gastroprotective effect of thymol is believed to be due to increased mucus secretion, prostaglandins and ATP-sensitive K^+^ channels (Ribeiro et al., 2016). Thymol (10–100 μM) induced the secretion of chloride (Cl^-^) and bicarbonate (HCO3^-^) ions in piglets’ intestinal epithelial cells via the nervous pathway likely through the activation of nervous nicotinic receptors (Boudry and Perrier, 2008). Thymol (0–100 μM) showed concentration dependent antispasmodic effects by inhibiting K^+^ induced tracheal contractions in rats (43% at 100 μM thymol). It also inhibits Barium chloride (BaCl_2_) induced tracheal contractions in a concentration dependent manner where the EC_50_ of thymol is in the range of 70 μg/mL. Also, Thymol (1.3 mg/kg) increased the mucociliary clearance in mouse trachea *in situ* (Begrow et al., 2010). Thymol (10 mg/kg) administered orally inhibited ethanol induced gastric mucosal injury by up-regulating the status of antioxidants and down regulating MMP-9 protein expressions (Chauhan and Kang, 2015). Thymol (0.072%) in 434 μg/ml extract of *Thymus vulgaris* concentration dependently antagonized the contractions in guinea pig trachea brought by four different spasmogens (Meister et al., 1999).

Thymol (IC_50_= 2.85 × 10^-2^± 1.2 × 10^-2^ μg/mL) isolated from *A. phleoides* essential oil has been shown to induce antispasmodic activity in charcoal meal mice (Astudillo et al., 2014). Thymol present in the extract of *Trachyspermum ammi* (0.01%) showed anti-spasmolytic and anti-spasmodic action against contractions in the isolated rat’s ileum induced by acetylcholine (Hejazian et al., 2014). Thymol (0–2 mM) exerted its relaxant effect on smooth muscle cells by opposing Ca^2+^ activation and ATP dependent process by its potent anti-spasmodic effect (Tamura and Iwamoto, 2004). Thymol (50 mg/kg) was shown to influence gastric maturation and function via stimulation of gastric proliferative activity and the control of digestive activity in weaned pigs (Colombo et al., 2014).

## Thymol In Models Of Liver Diseases

The protective effects of thymol in liver diseases are represented in **Table 4**.

### Hepatotoxicity

Thymol (30 mg/100 g) has been shown to inhibit oxidative stress in hydrocortisone-induced hepatotoxicity in rats by attenuating lipid peroxidation and enhancing antioxidant defense in the liver. Thymol treatment reinstated the activities of liver marker enzymes attributed to its potent free radical scavenging and antioxidant activity (Aboelwafa and Yousef, 2015). Thymol (300 mg/kg) has been shown to attenuate carbon tetrachloride induced liver injury in mice. Thymol treatment reduced lipid peroxidation and increased the status of antioxidants thereby preventing oxidative stress mediated hepatic injury in mice. Liver function tests and histological studies confirmed the other biochemical findings of the study (Al-Malki, 2010). In carbon tetrachloride (CCl_4_) (20 μl/kg) induced liver injury, thymol (300 mg/kg) abrogated lipid peroxidation and reinstated the normal activities of hepatic marker enzymes in the liver due to its potent free radical scavenging property (Alam et al., 1999).

Thymol (150 mg/kg) showed to inhibit paracetamol induced hepatotoxicity in mice by preventing the alterations in the activities of hepatic marker enzymes (Janbaz et al., 2003). Thymol (50 μg/ml) inhibited oxidative damage to liver cells by inhibiting ROS overproduction, ameliorating lipid peroxidation, preventing apoptosis and increasing antioxidant levels in tert-butyl hydroperoxide (t-BHP) induced Chang liver cells (Kim et al., 2014). Thymol (125 mg/kg) attenuated CCl_4_ induced hepatoxicity by inhibiting the release of glutamic pyruvate transaminase into the serum and it also decreased the levels of MDA in female Swiss OFFI mice (Jimenez et al., 1993). Thymol (1 ml/kg and 5.6 ml/kg) from thyme tincture and syrup inhibited CCl_4_ induced liver injury by reducing lipid peroxidation mediated oxidative stress and it maintained the levels of hepatic markers in Wistar rats (Raskovic et al., 2015). Thymol (50–200 mg/kg) increased the activities of phase I enzymes such as 7-ethoxycoumarin *O*-deethylase (ECOD) and phase II enzymes such as GST and quinone reductase (QR) along with raised activities of GST alpha and GST micro in mouse liver (Sasaki et al., 2005). In t-BHP induced Chang liver cells, thymol (50 μg/ml) inhibited lipid peroxidation and apoptosis by increasing the status of the antioxidants (Kim et al., 2014). These results revealed that thymol imparts a hepatoprotective effect on t-BHP-induced oxidative injury by mediating antioxidant activity (Kim et al., 2014). Thymol (25–100 μM) increased both enzymatic and non-enzymatic antioxidants and inhibited lipid peroxidation against paracetamol-induced toxicity in human HepG2 cells (Palabiyik et al., 2016).

## Thymol In Models of CNS Diseases

The protective effects of thymol in CNS diseases are represented in **Table 5**.

**Table 5:** Effect of thymol in different experimental models of neurogenerative disorders.

Dose	Model	Target/End points		Reference
		Increase	Decrease	
**Alzheimer’s disease**				
0.5–2 mg/kg	Wistar Rats	Aβ protein levels, cholinergic hypofunction	–	Azizi et al., 2012
0.30–25 μg/ml	PC-12 cells	Cell viability, antioxidant status	Oxidative stress	Lee et al., 2015
100 and 1000 μg/ml	PC-12 cells		AChE and BChE	Lee et al., 2015
**Anxiety**				
5, 10, and 20 mg/kg	Swiss albino mice	Time spent in open arms of elevated plus maze, percentage of time spent by mice in light compartment of light/dark test	–	Bhandari and Kabra, 2014
Aging				
42.5 mg/kg	Male Albino Wistar rats	SOD, GPx, total antioxidant status in the brain, phospholipid 18 : 2n-6, 20 : 1n-9, 22 : 4n-6 and 22 : 5n-3		Youdim and Deans, 1999
**Depression**				
15 and 30 mg/kg	CUMS	Sucrose consumption, body weight	Norepinephrine and serotonin (5-HT) in the hippocampus, IL-1β, IL-6, and tumor necrosis factor-α, NOD-like receptor protein 3, caspase-1	Deng et al., 2015
**Seizure**				
100 mg/kg 100 mg/kg	Male albino Wistar rats (MES model) Male albino Wistar rats (PTZ model)	– Prolonged onset of myoclonic jerk, onset of clonic seizure, onset of HLE, onset of death	Duration of HLE	Sancheti et al., 2014 Sancheti et al., 2014
100 mg/kg	Swiss albino mice (STR, model)	Prolonged the onset of death	Convulsions	Sancheti et al., 2014
25–100 mg/kg	Swiss albino mice		Locomotor activity	Sancheti et al., 2014
25 mg/kg	Swiss albino mice (PTZ, model)	Glutathione levels	Seizure score, MDA levels	Sancheti et al., 2014
10–50 mg/kg	Male albino Wistar rats (PTZ induced kindling model)	SOD	MDA, TNF-α and IL-1β expressions	Turrin and Rivest, 2004; Aliabadi et al., 2016
**Cholinergic dysfunction**				
10–100 ppm	*Caenorhabditis elegans* model	nAchR activity,	Synaptic Ach levels	Sammi et al., 2016
100–500 ppm	*Caenorhabditis elegans* model	Ache inhibition, acetylcholine esterase activity		Sammi et al., 2016
Thymol + gamma terpinene and thymol + para cymene (20 and 40 ppm)	*Caenorhabditis elegans* model	Synaptic Ach levels, nAchR activity		Sammi et al., 2016
**Other**				
0–1 mM	Mouse cortical neurons	Chloride influx		Garcia et al., 2006
1 mM	Rat spinal cord	Activation of TRPA1 channels, release of L-glutamate		Xu et al., 2015
2.7 mM (IC_50_= 0.34 mM)	CAP induced frog sciatic nerve	–	Voltage gated Na^+^ channels, peak amplitude	Kawasaki et al., 2013
200 μM/L (EC_50_ = 170 μM/L)	Rat neocortical slices	Release of GABA	Overflow of [H]-GABA	Parker et al., 2014
0–400 mg/L	N2a neuroblastoma cells	Cytotoxicity		Aydin et al., 2016

### Alzheimer’s Disease (AD)

Alzheimer’s disease is the most common cause of age associated dementia that leads decline in cognitive function following memory deterioration. Nowadays, treatment strategies have been developed for the management of AD with the use of acetylcholinesterase (AChE) inhibitors (an enzyme principally involved in the hydrolysis of acetylcholine) (Jukic et al., 2007). Thymol (EC_50_= 0.74 mg/mL) was shown to possess acetylcholine esterase inhibitory activity but much less than its isomer carvacrol (Jukic et al., 2007). In elderly patients, AD is associated with oxidative stress, inflammation and it is also characterized by the deposition of amyloid beta (Aβ) proteins in the central nervous system (CNS) which results in the formation of amyloid plaques, neurofibrillary tangles and area specific neuronal loss and synaptic changes in the brain (Duyckaerts et al., 2009). Thymol (0.5–2 mg/kg) has been shown to inhibit cognitive impairments caused by increased Aβ levels or cholinergic hypofunction in Aβ (25–35) or scopolamine treated rats attributed to its antioxidant, anti-inflammatory and anticholine esterase properties (Azizi et al., 2012). Thymol (0.39–25 μg/mL) has been shown to inhibit H_2_O_2_ induced oxidative stress in PC-12 cells whereas thymol (100 and 1000 μg/ml) also inhibited both AChE and butyrylcholinesterase (BChE) in a dose dependent manner (Lee et al., 2015).

### Anxiety

It is one of the most common mental disorders that is characterized by a disturbance in mood or emotional tone due to excessive fear. Thymol (5–20 mg/kg) has been shown to promote anti-anxiety activity in mice on both elevated plus-maze (EPM) and light/dark exploration test (LDT) behavioral models. This effect of thymol could be due to the possible modulation of the 5-hydroxytryptamine (5-HT), γ-aminobutyric acid (GABA) and nitric oxide-cyclic guanosine 3′,5′-monophosphate (NO-cGMP) pathways (Bhandari and Kabra, 2014).

### Dementia

Dietary supplementation of thymol (42.5 mg/kg) enhanced the status of endogenous antioxidants (SOD and GPx) and the proportion of PLs such as 18:2n-6, 20:1n-9, 22:4n-6 and 22:5n-3 in the aging rat brain (Youdim and Deans, 1999).

### Depression

Depression is a life threatening illness and the changes induced by inflammatory cytokines in monoamine neurotransmitters is a primary pathway of depression (Miller and Timmie, 2009). Thymol (15 and 30 mg/kg) has been shown to up regulate the levels of central neurotransmitters and inhibit the expressions of proinflammatory cytokines in unpredictable mild stress (CUMS) mice model (Deng et al., 2015).

### Epilepsy

Epilepsy is a devastating neurological disease characterized by spontaneous recurrent seizures affecting millions of people all over the world (Bhutada et al., 2010). Thymol (100 mg/kg) decreased the duration of the hind limb extension (HLE) in maximal electroshock (MES)-induced seizures. In pentylenetetrazole (PTZ)-induced seizure model, thymol (100 mg/kg) prolonged the onset of myoclonic jerk, onset of clonic seizures, onset of HLE and onset of death. Thymol (50 and 100 mg/kg) showed improved activity compared to diazepam in prolonging clonic seizure and the onset of myoclonic jerks. Furthermore, thymol (100 mg/kg) significantly prolonged the onset of death and reduced convulsions in the strychnine (STR) induced mouse model. Thymol (25 mg/kg, i.p.) significantly reduced seizure score, MDA levels and enhanced the levels of glutathione in the animal model of PTZ induced kindling (Sancheti et al., 2014). The authors revealed the antiepileptogenic potential of thymol by its Na^+^ channel blocking effect, positive modulation of GABA_A_ receptor and antioxidant property and they also concluded that it could be a potential candidate to treat epileptic patients (Sancheti et al., 2014). Thymol (10–50 mg/kg) attenuated PTZ (i.p. administration) induced epileptic stages in kindled rats via inhibiting oxidative stress markers in the serum MDA and with increased SOD activity. It also decreased the hippocampal pro-inflammatory cytokines viz. TNF-α and IL-1β released from astrocytes and microglia during and after the seizure induction in rats (Turrin and Rivest, 2004; Aliabadi et al., 2016). Thymol (ED_50_= 35.8 mg/kg) elicited inhibitory activity in the MES, sc Metrazol (scMET) and corneal-kindled models (Mishra and Baker, 2014).

### Cholinergic Dysfunction and Other Neurodegenerative Disorders

Cholinergic dysfunction is manifested in a plethora of neurodegenerative and psychiatric disorders such as Alzheimers, Parkinsons, and Huntington’s diseases. Thymol (10–100 ppm) in combination with gamma terpinene or para-cymene attenuated cholinergic dysfunction by enhancing synaptic levels of acetyl choline (Ach) and the responsiveness of nicotinic acetylcholine receptor (nAchR) in the *Caenorhabditis elegans* model (Sammi et al., 2016).

Thymol (100 μM) was shown to possess GABAergic activity and it potentiates GABAA-mediated inhibition of synaptic transmission *in vitro* (Marin et al., 2011). Thymol (0–1 mM) enhanced GABA-induced (5 mM) chloride influx at concentrations lesser than those revealing direct activity in the absence of GABA (EC_50_= 12 μM and 135 μM, respectively) in primary cultures of mouse cortical neurons (Garcia et al., 2006). A diet rich in thymol has been reported to enhance antioxidant defense and to maintain polyunsaturated fatty acid levels in aging rat brains (Youdim and Deans, 1999, 2000). Thymol has been reported to interact explicitly with synaptic neural functions and block the action of neuronal Na^+^ channels (Haeseler et al., 2002). Thymol raised the action of chloride channels in oocytes and the cell lines expressing GABA_A_ receptor subunits (Mohammadi et al., 2001; Priestley et al., 2003).

Recently, Sanchez et al. (2004) described the ability of thymol to integrate itself into the artificial membranes and enhance the binding affinity of (3H)flunitrazepam to GABA_A_ receptors in synaptosomal membranes that is indicative of thymol’s GABA_A_ receptor agonist/modulator property. Thymol (1 mM) has been shown to activate TRPA1 channels and increase the frequent release of L-glutamate on substantia gelatinosa (SG) neurons while generating an outward current without transient receptor potential (TRP) activation in adult rat spinal cord slices by its potent antinoceptive effect (Xu et al., 2015). Thymol (2.7 mM) (IC_50_= 0.34 mM) inhibited the peak amplitude in compound action potentials (CAP) in frog sciatic nerves (Kawasaki et al., 2013). Thymol (200 μM) potentiated the release of (3H)-GABA (EC_50_= 170 μM/L) probably by its antagonistic effect on GABA_b_ autoreceptors in rat neocortical slices (Parker et al., 2014). Thymol (0–400 mg/L) was shown to trigger cytotoxicity in N2a neuroblastoma cells (Aydin et al., 2016).

## Thymol In Lung Diseases

The protective effects of thymol in pulmonary diseases are represented in **Table 6**.

**Table 6:** Effect of thymol in different experimental models of pulmonary diseases.

Dose	Model	Target/End points		Reference
		Increase	Decrease	
**Asthma**				
4, 8 and 16 mg/kg	Female BALB/c mice	Goblet cells	Inflammatory cells, OVA-specific IgE, IL-4, IL-5, and IL-13, AHR, mucous hypersecretion, inflammatory infiltrates, mucus hypersecretion and goblet cell hyperplasia, IκB, p-IκB-α, p65 and p-p65 expression	Zhou et al., 2014
0.7 μg/ml/kg	Male Wistar albino mice	Hemoglobin, SOD and GPx	NO, H_2_O_2_, MDA, Isoprostane, carbonyl group	Al-Khalaf, 2013
50 mg/kg	Ova-Alum induced asthmatic rats	SOD, catalase and GSH	Oxidized glutathione	Mottawie et al., 2011
100, 200, and 400 mg/kg	OVA-induced rodents	–	Cough, tracheal fluid volume	Ozolua et al., 2016
80 mg/kg	OVA induced male BALB/c mice and cultured spleenocytes	mRNA levels of IL-10, TGF-β	Foot pad thickness, spleenocyte cell proliferation, mRNA levels of IFN-γ, IL-4, IL-5, IL-17A, IL-23, T_H_1 cytokine (IL-2 and IFN-γ), T_H_2 (IL4), T_H_17 (IL-17A) levels, IL-4 formation, IL-17 secretion, T-box 21 (T-bet) expression, GATA binding protein 3 expression, RAR-related orphan receptor C	Gholijani and Amirghofran, 2016
200 μM or 30 μg/mL (Thymol and carvacrol)	BEAS-2B cells	SHIP1 and SOCS1 mRNA and protein levels	Levels of IL-25, IL-33, TLR2, TLR4 expression, induction of miR-155 and miR-21 and completely prevented the induction of miR-146a	Khosravi and Erle, 2016
**Pleurism**				
750 mg/kg	Male Wistar rats	–	Inflammatory edema, migration of leucocytes	Fachini-Queiroz et al., 2012

### Asthma

Asthma is an inflammatory disorder characterized by the infiltration of inflammatory cells into lung tissues, hypersecretion of the mucus by goblet cells, airway hypereactivity (AHR), Th2 mediated cytokines and their over-expressions including IL-4, IL-5 and interleukin-13 (IL-13) (Rogerio et al., 2010). Thymol (4, 8, and 16 mg/kg) has been shown to abrogate hyperresponsiveness (AHR) and allergic airway inflammation by attenuating infiltration of inflammatory cells, Th2 cytokines and ovalbumin (OVA)-specific IgE and suppressing the pathological changes due to its NF-κB activation blocking property in OVA-induced allergic mice (Zhou et al., 2014). Thymol (0.7 μg/ml/kg) attenuated ovalbumin induced bronchial allergic asthma by inhibiting oxidative stress in male Wistar albino mice (Al-Khalaf, 2013). In OVA induced mice, thymol (80 mg/kg) suppressed the antigen-specific immune response by inducing reductions T_H_ cells [T_H_1, T_H_2 and T-helper cell 17 (T_H_17)]-related cytokines and key transcription factors, revealed their potential to modulate over-activation of T-cells and the associated destructive immune responses (Gholijani and Amirghofran, 2016). Thymol (50 mg/kg) attenuated oxidative stress mediated bronchial asthma in OVA-Alum induced rat erythrocytes by increasing the status of antioxidants (Mottawie et al., 2011). These findings suggest that thymol possesses the potential to be used as an agent for therapeutic benefits in asthma. However, for the clinical usage, comprehensive safety and efficacy studies are further required (Zhou et al., 2014).

Thymol present in the leaf extract of *Ocimum gratissimum* Linn (100, 200, and 400 mg/kg) suppressed coughing in OVA induced bronchial asthma by reducing tracheal fluid secretion in rodents through its anti-asthmatic and antitussive effects (Ozolua et al., 2016). A previous report from Gavliakova et al. (2013) has revealed that nasal administration of thymol has been associated with the reduction of cough in asthma patients by an olfactory mechanism. Intake of one bronchipret (around 1.08 mg of thymol) for about a month improved the compliance, pulmonary pressure and airway resistance in the lungs of horses (Van den Hoven et al., 2003). Thymol at higher concentrations (10^-4^–10^-2^ M) showed bronchodilatory effects in guinea-pig tracheal preparations (Astudillo et al., 2014). Combined treatment with carvacrol/thymol (200 μM, equal to 30 μg/mL) inhibited the effects of chitin induced asthma by suppressing type 2-promoting release of cytokines and Src Homology 2 (SH2) domain-containing inositol polyphosphate 5′ phosphatase 1 (SHIP1), toll like receptors (TLRs), cytokine signaling 1 (SOCS1) and micro RNAs expressions. It also reduced the toll like receptor 4 (TLR4), toll like receptor 2 (TLR2) protein levels and increased the SHIP1 and SOCS1 protein levels (negative regulators of total knee replacement (TKR) mediated immune response) in immortalized human bronchial epithelial cells (BEAS-2B cells). This study revealed the inhibitory effects of carvacrol/thymol treatment against chitin induced epithelial cell pro-inflammatory responses (Khosravi and Erle, 2016).

### Pleurism

Thymol (750 mg/kg) has been shown to abrogate carrageenan induced pleurisy by inhibiting the accumulation of inflammatory exudates in the pleural cavity of the lungs (Fachini-Queiroz et al., 2012).

## Thymol In Radiation Induced Toxicity

The protective effects of thymol in radiation-induced toxicity are represented in **Table 7**. Radiotherapy for the treatment of various cancers has been shown to induce serious damage to both tumors and normal cells. A report from Archana et al. (2011b) has revealed that thymol (0–100 μg/mL) diminished radiation-induced genotoxicity, apoptosis and necrosis in V79 cells primarily by the free radical scavenging and modulation of oxidative stress. Thymol treatment prevents the collapse of mitochondrial membrane potential (MMP) and protects the cells from apoptotic and necrotic cell death (Archana et al., 2011b). The radioprotective and anticlastogenic potential of a phenol derivative monoterpene thymol has been reported in whole-body gamma radiation induced Swiss albino mice (Archana et al., 2011a). The antioxidant, anticlastogenic and radioprotective potential of thymol is attributed to the stabilization of intracellular antioxidant levels and free radical scavenging activities by thymol (Archana et al., 2011a). The radioprotective potential of thymol is also demonstrated by increased LD50/30 by 2.17 gray (Gy) which resulted in a dose reduction factor (DRF) of 1.25 (Archana et al., 2011a). Thymol (5 μg/ml) has been shown to abrogate radiation induced cytotoxicity by inhibiting the levels of lipid peroxidation and increasing the status of antioxidants in V79 cells grown *in vitro* (Archana et al., 2011a). Thymol (1 μg/ml) inhibited UV radiation A (UVA) and UV radiation B (UVB) induced genotoxicity via inhibiting oxidative stress in the NCTC 2544 cell line (Calo et al., 2015). Thymol (1 μg/ml) attenuated UV radiation induced genotoxic damage in *ex vivo* human skin models by its potent anti-cancer properties (Cornaghi et al., 2016).

**Table 7:** Effect of thymol on radiation toxicity.

Dose	Model	Target/End points		Reference
		Increase	Decrease	
**Gamma radiation**				
Thymol (0–100 μg/mL) + 3 Gy gamma irradiation	V79 cells	Mitochondrial membrane potential	Micronuclei, DNA double strand breaks, percentage of tail DNA, apoptosis, necrosis	Archana et al., 2011b
Thymol (10 mg/kg) + gamma radiation (4.5 and 7.5 gy)	Swiss Albino mice	GSH, GST, catalase, SOD, white blood cells (WBC) count, red blood cells (RBC) count, number of spleen colonies	Micronucleated polychromatic erythrocytes and micronucleated normochromatic erythrocytes, MDA	Archana et al., 2011a
5 μg/ml	V79 cells	Cell viability, glutathione, SOD and catalase	ABTS, DPPH, superoxide anion, hydroxyl radicals, apoptosis, DNA fragmentation, intracellular ROS, lipid peroxidation levels	Archana et al., 2011a
**UV radiation**				
1 μg/ml	NCTC 2544 cells	Nucleotide excision repair expressions	ROS, MDA, DNA double strand breaks	Calo et al., 2015
1 μg/ml	*Ex vivo* human skin tissue model	Scattered H2AX-positive cells	LDH release, % DNA-Tail	Cornaghi et al., 2016

## Thymol In Autoimmune Diseases

The protective effect of thymol against autoimmune diseases is represented in **Table 8**. Rheumatoid arthritis, an autoimmune disease is characterized by chronic and progressive inflammation of the synovial joints and erosive destruction of the articular tissue (Feldmann et al., 1996; Feldmann, 2002; Choi et al., 2009). Thymol (100 mg/kg) was shown to inhibit collagen induced arthritis by decreasing lipid peroxidation mediated oxidative stress by increasing the status of antioxidants in male Wistar rats. Thymol also stopped the activity of elastase, a marker for collagen degradation and prevented the invasion of inflammatory cells to the injured site by blocking the Ca^2+^ channels (Braga et al., 2006; Umar et al., 2012). The physical mixture of diacerein and thymol (DTH) (50 + 20.4 mg/kg) abrogated Freund’s complete adjuvant (FCA) induced arthritis in male albino Wistar rats. This combined treatment decreased oxidative stress, ulcer index and synovitis in arthritic rats by its potent antioxidant property. DTH administration also improved the histoarchitecture as evidenced by decreased necrosis in bones, cellular infiltration, connective tissue proliferation and the involvement of adjacent tissues (Dhaneshwar et al., 2013). In Jurkat leukemia cells as an *in vitro* T cell model, thymol (25 μg/ml) modulated T-cell activity by reducing IL-2 and IFN-γ production via down regulation of AP-1 and nuclear factors of activated T-2 (NFAT-2) transcription factors showing its capacity in reducing the overactivity of T-cells in immune mediated diseases (Gholijani et al., 2015).

**Table 8:** Effect of thymol in different experimental models of autoimmune diseases, reproductive and metal induced toxicity.

Dose	Model	Target/End points		Reference
		Increase	Decrease	
**Rheumatoid arthritis**				
100 mg/kg	Male Albino Wistar rats	SOD, catalase, GSH,	TBARS, NO, release of elastase, Ca^2+^ channels	Braga et al., 2006; Umar et al., 2012
**Osteoarthritis**				
Diacerein and thymol (50 + 20.4 mg/kg)	Male albino Wistar rats (Rainsford’s cold stress model)	Lipophilicity, bioavailability, absorption	Oxidative stress, edema, ulcer index, synovitis, cellular infiltration, bone necrosis, connective tissue proliferation, adjacent tissue involvement	Dhaneshwar et al., 2013
**Other**				
25 μg/ml	Jurkat leukemia cells	-	Levels of IL-2, IFN-γ, NFAT-2, c-FOS	Gholijani et al., 2015.
**Male infertility**				
400 mg/kg	Male albino Wistar rats	Abnormal sperms	Sperm count, motility, testis weight	Surendra Kumar et al., 2011
100–500 μg/ml	Human spermatozoa	Abnormal sperms	Sperm count, sperm motility and vitality,	Chikhoune et al., 2015
**Chromium induced toxicity**				
2.5 μg/ml	Isolated rat erythrocytes	SOD, catalase, GSH	MDA, hemolysis, erythrocyte destabilization	Abd-Elhakim and Mohamed, 2016
**Arsenic and mercury induced toxicity**				
0–200 μM/L	Male Wistar rats	Resting tension and mean relaxation of aorta and trachea	Aortic and tracheal contractions, ROS, Ca^2+^ influx	Kundu et al., 2016
100 μM	Hgcl_2_ induced HepG2 cells	Cell viability, mitochondrial membrane potential, SOD, catalase and GSH levels	Mirconucleated binucleated cell frequency, micronucleous frequency, percentage of tail DNA, DNA damage, apoptosis, necrosis, ROS generation, superoxide radicals, MDA levels	Shettigar et al., 2015

## Thymol In Reproductive Disorders

The protective effects of thymol in reproductive disorders are represented in **Table 8**.

### Contraceptive

Male infertility refers to the inability of males to cause pregnancy in females usually due to reduced sperm quantity and quality (Cooper et al., 2009). Thymol (400 mg/kg) decreased fertility in male albino Wistar rats. Thymol decreased the weight of testis, sperm count and motility and increased the amount of abnormal sperms in rat testis (Surendra Kumar et al., 2011). Chikhoune et al. (2015) revealed the anti-fertility effect of thymol in human spermatozoa. Thymol (100–500 μg/ml) dose dependently decreased sperm count, sperm motility, sperm vitality in human sperm. These two studies have revealed that thymol could be used as a standard contraceptive agent in humans.

## Thymol In Metal Induced Toxicity

The protective effects of thymol in metal induced toxicity are represented in **Table 8**.

### Chromium

Chromium is a naturally occurring, highly toxic transition metal due to its strong ability to oxidize cellular components through its passive entry via cellular membranes into cells (O’Brien et al., 2003). Thymol (2.5 μg/ml) has been shown to inhibit hexavalent chromium induced oxidative damage in rat erythrocytes. Thymol treatment significantly decreased MDA levels, hemolysis, erythrocyte destabilization and increased the activities of antioxidants enzymes and improved the levels of glutathione in rat erythrocytes (Abd-Elhakim and Mohamed, 2016).

### Arsenic and Mercury

Arsenic and mercury are toxic metals found in nature in soil, in industrial and agrochemicals as well as pharmaceuticals. Upon exposure, they are known to cause acute and chronic disease and mainly affect smooth muscles of the cardiovascular and respiratory systems. Thymol (0–200 μM/L) abrogated arsenic and mercury induced hyper contraction of both aortic and tracheal smooth muscles by inhibiting Ca^2+^ influx at low concentrations. It also neutralizes ROS and inhibits Ca^2+^ influx at higher concentrations (Kundu et al., 2016). Thymol (100 μM) was shown to protect against cytotoxicity and genotoxicity induced by mercuric chloride in the human HepG2 cell line due to its potent free radical scavenging ability that in reflected in the attenuation of mitochondrial and oxidative damage (Shettigar et al., 2015). Thymol present in the essential oil of *T. lanceolatus* (IC_50_= 256.17 μg/ml) was shown to induce cytotoxicity and cell proliferation in HepG2 cells (Khadir et al., 2016).

## Pharmaceutical Development of Thymol

Nowadays, the focus on natural products is to develop their formulation with improved bioavailability, favorable pharmacokinetics and minimal adverse effects. Various attempts have also been made to develop thymol formulation with improved drug delivery options for the treatment of various human diseases. The pharmacokinetic and physiochemical properties of thymol including absorption, bioavailability, elimination rate, solubility are the major barricades in the drug design and delivery of thymol. There are lot of techniques such as structural modification (Mastelic et al., 2008), microparticles using cellular derivatives (Zamani et al., 2015), encapsulation (Rassu et al., 2014), solid dispersion (Roost et al., 2015), complexations (Nieddu et al., 2014) and nanoparticle formulation (Pan et al., 2014; Zhang et al., 2014) which could pave the way to advance drug delivery options for thymol and these are mentioned below.

## Structural Modification For Future Drug Development

The structural alterations into the phenol structures, like introducing a polar hydroxymethyl moiety, could enhance its antioxidant activity compared to parent compounds (Torres de Pinedo et al., 2007). The derivative of thymol, named 4-(hydroxymethyl)-2-isopropyl-5-methylphenol, was synthesized by the hydroxymethylation of thymol (Mastelic et al., 2008). The phenolates as nucleophiles reacted with methanol which yielded hydroxymethylphenols at alkaline pH. This might be due to the delocalization of the phenolate ion charge between the phenolate oxygen and its respective ortho and para- carbons. The steric effect of the isopropyl group was believed to confer improved antioxidant activity and reduced mitochondrial activity of thymol derivative in HeLa cells in a concentration dependent manner (Mastelic et al., 2008).

A set of new thymol derivatives invented and patented recently showed potent antitumor activity against A549, SKOV-3, human melanoma cells (SK-MEL-2), cellosaurus cells (XF498) and colorectal adenocarcinoma cells (HCT15 cells) (Zee et al., 2001). Thymol analogues such as 4-morpholinomethyl-2-isopropyl-5-methylphenol (THMO) and 4-Pyrrolidinomethyl-2-isopropyl-5-methylphenol (THPY) were synthesized by the reaction between thymol and formaldehyde with morpholine or pyrrolidine (Shen et al., 2005). These two analogs of thymol showed a potent superoxide anion scavenging effect *in vitro* and in human blood neutrophils and also possess a superior lipid peroxidation inhibitory effect via the attenuation of enzymes involved in antioxidant defense. In the two thymol analogs, THMO revealed potent antioxidant activity with IC_50_ values of 21.72 and 61.29 μM for the inhibition of xanthine oxidase and lipid peroxidation (Shen et al., 2005). THMO (10 mM) also decreased the peak amplitude of L-type inward current of Ca^2+^ (I_Ca,L_) in NG108-15 cells as analyzed by the patch-clamp technique. These reports have revealed that the antioxidative action of the thymol analogs is linked with its capacity of inhibiting Ca^2+^ current (Shen et al., 2005). This study suggests that THMO could be a suitable candidate for the treatment of free radical related disorders by virtue of its antioxidant and Ca^2+^ ion current inhibition activity.

## Microencapsulation For Drug Delivery

Microencapsulation is a tool frequently used in pharmaceutical, food, cosmetic, and agrochemical industries. The encapsulation of thymol in microspheres made up of natural polymers such as methylcellulose and hydroxylpropyl methylcellulose phthalate can serve to obtain efficient delivery of this phytochemical as adjuvants or current medications for the treatment of infectious diseases and compensate the limited bioavailability due to its lower solubility (Rassu et al., 2014). The core-shell or matrix particle encapsulation of essential oils has been investigated to determine their controlled release (Martins et al., 2014). The encapsulation of thymol into methylcellulose microspheres by spray drying remarkably increases the bioavailability compared to free thymol and it can be suggested to be used for the treatment of intestinal infections (Rassu et al., 2014).

The synthetic, natural and semisynthetic polymers play a crucial role in drug release formulations and nowadays these are used as efficient drug carriers (Nayak et al., 2009). Cellulose derivatives have been used for sustained release matrices, delayed release dosage forms, binders in granules and tablets and they also have many other applications (Chambin et al., 2004). Zamani et al. (2015) has structured the matrix polymer encapsulation of thymol by the emulsion solvent evaporation method with hydroxy propyl methyl cellulose (HPMC) and ethyl cellulose (EC) to increase the duration of action of thymol. Both polymers have shown a considerable effect on drug release behavior, efficiency of drug entrapment, drug loading and particle size, whereas the formulation F6 revealed a controlled effect compared to the other formulations in *in vitro* release (Zamani et al., 2015).

## Co-Administration For Drug Delivery

A co-drug DTH has been developed recently by Dhaneshwar et al. (2013). Diacerein, an IL-1β inhibitor possesses anti-arthritic and moderate anti-inflammatory properties (Dhaneshwar et al., 2013). This mutual prodrug was developed by the covalent linkage of thymol with the carboxylic acid (-COOH) group of diacerein and this prodrug showed lessened irritant effect, improved absorption, prolonged drug release with improved anti-inflammatory effect. The hydrophobic nature of thymol enhanced the lipophilicity of diacerein which could be responsible for its enhanced bioavailability and its better absorption. The synthesis of DTH was done by the dicyclohexylcarbodiimide (DCC) coupling method (Holmberg and Hansen, 1979) and its physicochemical characterization was evaluated using spectral analysis. DTH was very stable in acidic pH conditions of the stomach and complete diacerein release was observed in phosphate buffer (91–94%) (pH 7.4) and in the small intestine.

Thymol has been used as an antioxidant agent in this design and its selection was justified after the promising anti-inflammatory and anti-arthritic effect of DTH against osteoarthritis compared to standard drugs which is attributed to the pharmacological effects of thymol. DTH attenuated FCA induced chronic synovitis in rats by its marked antiarthritic effect compared to the moderate effect of diacerein and mild effect of thymol. The authors suggested that the combination of diacerein with thymol could be promising therapeutic approach in inflammation related diseases (Dhaneshwar et al., 2013).

## Concluding Remarks

The need for alternative therapies with less toxic effects for various human ailments is evident. The findings from various studies reviewed herein showed the role of thymol in the prevention of various types of diseases through its multi-pharmacological properties from antioxidant to anti-tumor ones. Thymol containing plants have been used in traditional medicine for management of various diseases such as many cancer types, cardiovascular diseases, diabetes, and neurodegenerative diseases. Multiple pharmacological and molecular mechanisms of action for its preventive and therapeutic effects have been demonstrated based on its molecular targets identified in numerous studies. While a great number of *in vitro* studies for numerous diseases including cancer and cardiovascular diseases have been reported, more *in vivo* studies should be undertaken to confirm the *in vitro* findings. In addition, there is a contradiction between *in vitro* concentrations and *in vivo* doses in certain types of cancer. Thus, pharmacokinetics and pharmaceutical studies are needed to interpret the inconsistency between *in vitro* and *in vivo* results. These reported features along with the minimal side effects, cost effectiveness and easy access made thyme and its constituent thymol an effective therapeutic agent for the management of numerous chronic diseases. Furthermore, thymol, being abundantly and ubiquitously present in numerous plants, could be available for dietary use. Its administration and benefits could be achieved in a simpler way through normal daily diet. However, the vast majority of the data is preclinical, and further clinical studies are warranted. Furthermore, comprehensive toxicological studies should be conducted to support the safety of thymol in animal models to progress for clinical studies. Though, taking together all the studies, it is significant to say that research on thymol as a drug candidate is progressive and encouraging. This has been well demonstrated by the publication patterns year after year. Hence, thymol is one of the most powerful contenders in the race of phytochemicals of natural origin with polypharmacological properties displaying potent preventive and therapeutic properties against various human diseases.

## Author Contributions

SO and MN conceptualized and outlined the study. MN drafted the manuscript. HAT, SA, HJ, and SO edited and reviewed the manuscript. SO and MN throughly re-reviewed it and all authors approved it.

## Conflict of Interest Statement

The authors declare that the research was conducted in the absence of any commercial or financial relationships that could be construed as a potential conflict of interest. The reviewer FPG and handling Editor declared their shared affiliation, and the handling Editor states that the process nevertheless met the standards of a fair and objective review.
